# Identification of Mur34 as the Novel Negative Regulator Responsible for the Biosynthesis of Muraymycin in *Streptomyces* sp. NRRL30471

**DOI:** 10.1371/journal.pone.0076068

**Published:** 2013-10-15

**Authors:** Dongmei Xu, Guang Liu, Lin Cheng, Xinhua Lu, Wenqing Chen, Zixin Deng

**Affiliations:** 1 State Key Laboratory of Microbial Metabolism, and School of Life Sciences & Biotechnology, Shanghai Jiao Tong University, Shanghai, China; 2 Key Laboratory of Combinatorial Biosynthesis and Drug Discovery (Wuhan University), Ministry of Education, and Wuhan University School of Pharmaceutical Sciences, Wuhan, China; 3 NCPC New Drug Research and Development Co., Ltd, North China Pharmaceutical Group Corporation, Shijiazhuang, China; Baylor College of Medicine, United States of America

## Abstract

**Background:**

Muraymycin, a potent translocase I (MraY) inhibitor, is produced by *Streptomyces* sp. NRRL30471. The muraymycin gene cluster (*mur*) was recently cloned, and bioinformatic analysis of *mur34* revealed its encoding product exhibits high homology to a large family of proteins, including KanI and RacI in individual biosynthetic pathway of kanamycin and ribostamycin. However, the precise role of these proteins remains unknown.

**Principal Findings:**

Here we report the identification of Mur34 as the novel negative regulator involved in muraymycin biosynthesis. Independent disruption of *mur34* on chromosome and cosmid directly resulted in significant improvement of muraymycin production by at least 10 folds, thereof confirming the negative function of Mur34 during muraymycin biosynthesis and realizing the engineered production of muraymycin in heterologous host. Gene expression analysis indicated that the transcription level of the *mur* genes in *mur34* mutant (DM-5) was dramatically enhanced by *ca*. 30 folds. Electrophoretic mobility shift assay (EMSA) showed that Mur34 specifically bound to the promoter region of *mur33*. Further experiments showed that a 28-bp region downstream of the transcription start point (TSP) was protected by His_6_Mur34, and the −10 region is essential for the activity of *mur33* promoter.

**Conclusions:**

Mur34 plays an unambiguously negative role in muraymycin biosynthesis *via* binding to the upstream of *mur33*. More importantly, Mur34 represents a novel family of regulators acting in negative manner to regulate the secondary metabolites biosynthesis in bacteria.

## Introduction

*Streptomycetes* are usually soil-living organisms with complex life cycle that includes formation of aerial mycelia and spores. Members of this genus have relatively large genomes and the capability of producing tremendous number of secondary metabolites, many of which have been used as antibiotics, anti-tumor agents, and immunosuppressants [1].

**Figure 1:**
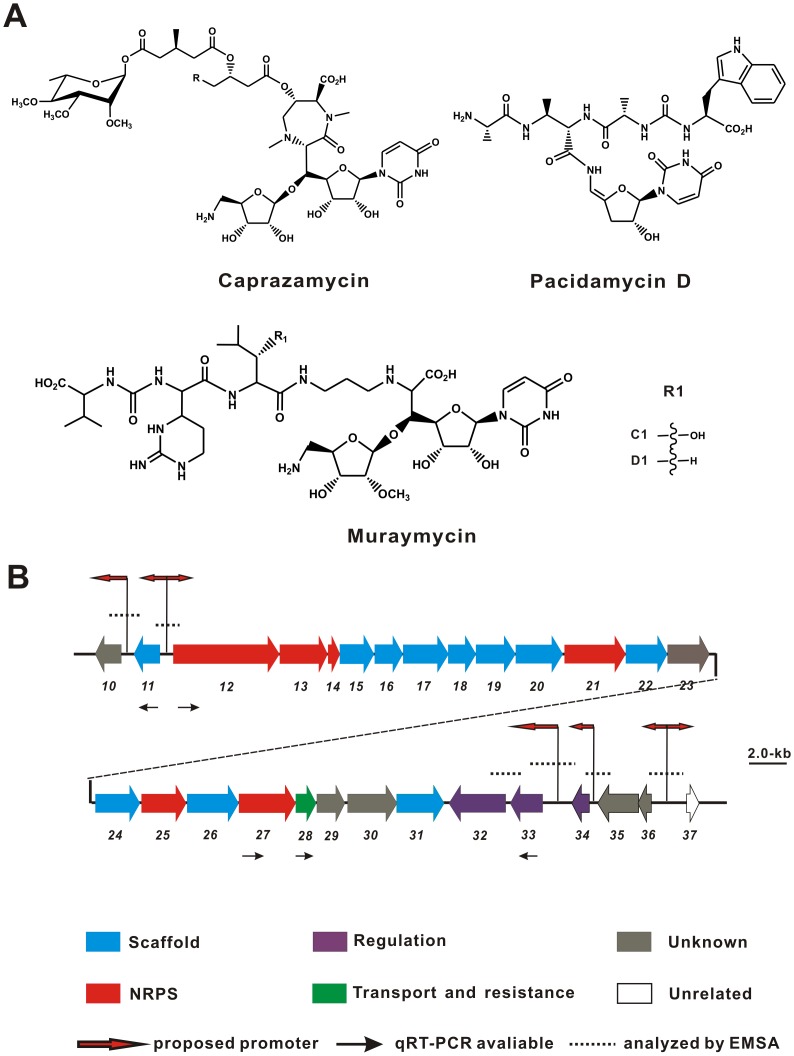
Chemical structure of related antibiotics (A) and genetic organization of the muraymycin gene cluster (B). Red arrows indicate the promoters which are probably the approximate binding sites of Mur34, black arrows mean the specific genes selected for quantitative real-time PCR, and the dotted lines show the gene intergenic regions analyzed by EMSA.

Muraymycins, a group of structurally related nucleoside antibiotics, are powerful translocase I (MraYs) inhibitors. This family of antibiotics including well-characterized pacidamycin and caprazamycin was recently pursued for their unusual structures and outstanding bioactivity with clinic potential [2] (Fig. 1A). As a competitive translocase I inhibitor, muraymycin targets bacterial cell wall biosynthesis by inhibiting the activity of phospho-UDP-N-acetylmuramoyl-pentapeptide translocase (MraY, translocase I) which catalyzes at an early stage of peptideglycan biosynthesis, as a result, muraymycin leads to the bacteria a loss of cell shape and integrity followed by cell death [3]–[4]. Distinctively, muraymycin was prevalently recognized as a novel promising lead-chemical for its amenable structure and the typical scaffold, and the pioneer semisynthesis of their structures was initiated by Lin *et al* [5] and Matsuda *et al* [6].

*Streptomyces* was distinguished for its large and complex regulation system in the biosynthesis of antibiotics. The well-known “microbial hormones” γ-butyrolactones play an important role in the secondary metabolite regulation systems [7]–[8], and many γ-butyrolactones binding to their receptors are involved in the regulation of specific antibiotic biosynthesis. As exemplified by ArpA, the receptor protein of A-factor belonging to the TetR family, functions as a repressor responsible for the production of streptomycin, grixazone and other secondary metabolites [9]. Most of the butyrolactone receptors are autoregulators, which usually locate close to the antibiotic biosynthesis genes [10]. This family of regulators, involving FarA which is in charge of the biosynthesis of nucleoside antibiotics, minimycin and showdomycin in *S. lavendulae* FRI-5 [11]–[12], is widely distributed in *Streptomyces* [13], Structurally, most of the regulators are dimmers, e.g. CprB, a TetR family regulator from *S. coelicolor*, whose structure includes an HTH domain and a hydrophobic cavity which is probably the ligand-binding pocket [14].

The second different mechanism for the regulation of secondary metabolite biosynthesis is exerted by two-component systems (TCSs) involving the previously described AbsA1-AbsA2 in *S. coelicolor* [15]–[16]. For AbsA1-AbsA2, the phosphorylated form of AbsA2 acts as negative regulator for antibiotic production [17]. Moreover, as for the well reported PhoR-PhoP two-component system in *S. lividan*s, *S. natalensis* and *S. coelicolor*, the responser phosphorylated PhoP as a negative regulator controls the downstream gene transcription by binding to the well-known PHO box, which was composed of direct repeats units (DRus) [18]–[21].

The muraymycin gene cluster was previously identified and characterized from *Streptomyces* sp. NRRL 30471. Systematic analysis of the whole gene cluster indicated that one potential regulatory gene *mur34* was proposed to be involved in the regulation of muraymycin production [22], whereas little is known for the regulatory mechanism of muraymycin biosynthesis. Here we report the identification and characterization of Mur34 as an atypical negative regulator with widespread distribution in bacteria, which would lay a solid foundation for deeper understanding of such regulatory mechanism in secondary metabolites biosynthesis, and also be helpful for rational enhancement of target antibiotics production *via* synthetic biology strategies.

## Results

### In silico analysis of mur34

*mur34* encodes a protein of 158 amino acids with a calculated molecular mass of 17.5 kDa, and the secondary structure of Mur34 includes 55.3% helixes and 44.7% loops predicted by PredictProtein software (http://www.predictprotein.org/) [23]. BlastP analysis of Mur34 shows that it displays significant homology to LivI (74% identities), RacA (76% identities), and KanI (74% identities) which are correspondingly involved in the biosynthesis of lividomycin, ribostamycin and kanamycin (Fig. S1, A), while the precise function of the proteins remains unraveled. Further analysis by SMART online program (http://smart.embl-heidelberg.de/) indicates that Mur34 belongs to an all-alpha protein class and λ repressor-like DNA-binding domains superfamily with an e-value of 7.00e-04, implicating that Mur34 probably functions as a regulator involved in muraymycin biosynthesis.

### Mutation and complementation of mur34

**Figure 2:**
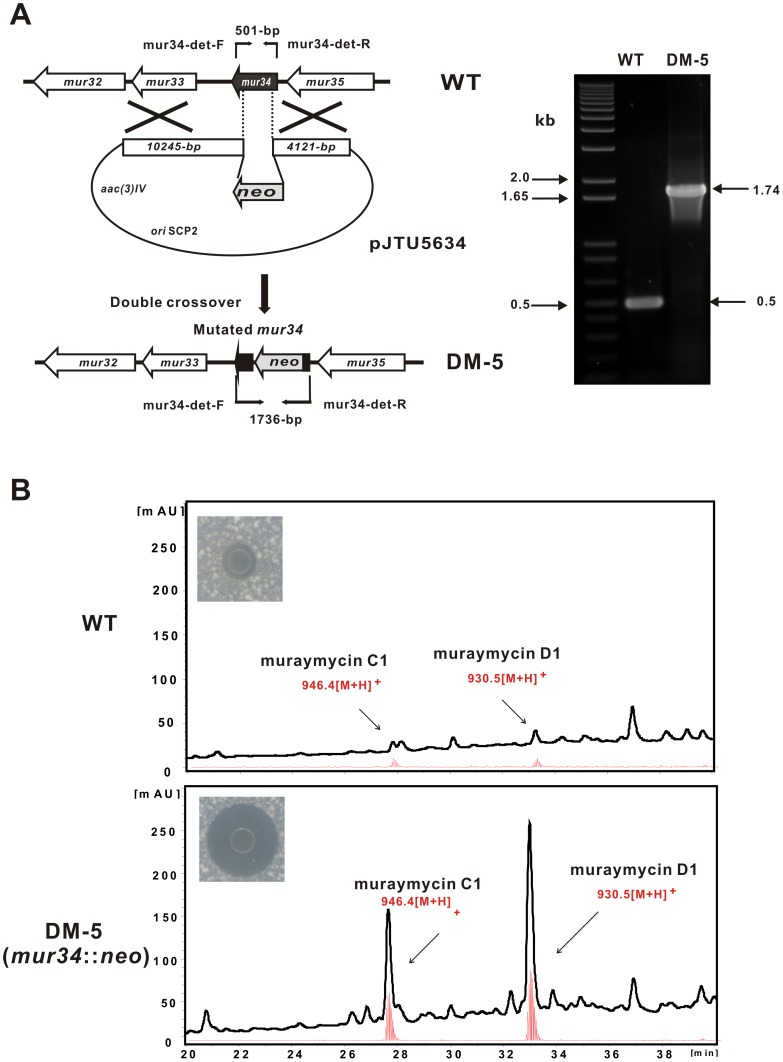
Mutational analysis of *mur34*. (A) Schematic representation for the construction of DM-5 mutant, as a 0.23-kb of *mur34* was replaced by the 1.43-kb *neo* cassette, the DM-5 mutant gives a 1.74-kb PCR product, while the wild type strain is 0.5-kb. (B) Bioassay and LC-MS analysis of the metabolites. Top, the metabolites produced by wild type strain. Bottom, the metabolites produced by DM-5 mutant. Muraymycin C1 and D1 components were selected for LC-MS comparative analysis.

To establish if Mur34 could play a regulatory role in the biosynthesis of muraymycin, the *mur34* disruption vector pJTU5634 was introduced into *Streptomyces* sp. NRRL30471 *via* conjugation. According to the standard strategies [24], the candidate mutant (Apr^S^Neo^R^) was selected for further PCR confirmation. Corresponding to expectation, genomic DNA sample of the randomly selected candidate could produce the 1.74-kb PCR product, whereas that of the wild type (WT) strain gives the 0.5-kb product, indicating that *mur34* was successfully mutated (Fig. 2A, B). To further investigate the phenotype of *mur34*, the mutant (designated as DM-5) was inoculated for fermentation, and the broth samples were then purified and submitted for bioassay and liquid chromatography-mass spectrometry (LC-MS) analysis. Results showed that the DM-5 sample harbors more potent bioactivity against the indicator strain than that of the WT strain (Fig. 2C). Further LC-MS analysis revealed that the muraymycin productions (C1 and D1 components) of the DM-5 fermentative sample were apparently improved by 10-fold as compared with that of the wild type strain, which is consistent with the bioactivity of muraymycin against the indicator strain (Fig. 2C). The production of muraymycins was almost inhibited in DM-5 by introducing *mur34* under its natural promoter (Table S1 in File S1) (Fig. S2). All these results distinctly suggested that Mur34 acts as a negative regulator involved in muraymycin biosynthesis.

### Utilization and inactivation of mur34 in 18F3 cosmid for heterologous expression and determination of the minimal muraymycin gene cluster

As 18F3 was deduced to cover the whole gene cluster of muraymycin, it was introduced into *S*. *lividans* TK24 for heterologous production of muraymycin, however, the components of muraymycins almost could not be detected by LC-MS and bioassay. For further engineered production of muraymyicns, *mur34* in 18F3 cosmid was inactivated *via* PCR-targeting strategy [25], the resultant cosmid pJTU5642 (Fig. S3, A) was introduced into *S. lividans*. Results showed that the production of the antibiotics by the recombinant strain (TK24/pJTU5642) (Table S1 in File S1) was detected and increased dramatically (Fig. S3, B), suggesting that 18F3 harbors the entire gene cluster of muraymycin and *mur34* contributes to the significant enhancement of muraymycin production.

In order to further investigate the minimal gene cluster of muraymycin, two target genes were mutated in pJTU5030 (Fig. S3, A), which is a derivative of pJTU5642. The biosynthesis of muraymycins could not be affected after disruption of *mur11* (pJTU5024) (Fig. S3, C) (Fig. S3, D). However, disruption of *mur12* (pJTU5053) abolished the antibiotics production (Fig. S3, E) (Fig. S3, F). The results indicated that *mur12* is essential for the biosynthesis of muraymycins, consistent with the speculation that Mur12 is necessary in the activation of amino acid substrate[22]. As a result, the minimal gene cluster of muraymyicn was pinpointed as the genes involving *mur12*-*mur34*.

### Gene transcription analysis of the mur genes in DM-5 (mur34::neo) strain

**Figure 3:**
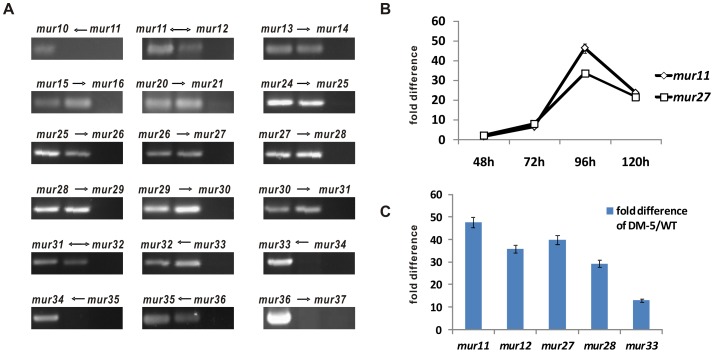
Gene expression analysis of the *mur* genes. (A) Transcription analysis of intergenic region of the selected *mur* genes. Top, ethidium bromide-stained agarose gels showing RT-PCR fragments from intergenic regions. *mur10*←*mur11* means that the detected region between *mur10* and *mur11*, and the arrows showed the possible orientation of transcription. In each gel, the left band was positive control using genomic DNA as template, the middle band showed the PCR sample using cDNA as template, the right band is negative control using template from total RNA sample digested with DNase I. (B) Time course of the transcription difference of *mur11* and *mur27* for DM-5 and the wild type strain. (C). The transcription difference of DM-5 and the wild type strain for 96 h incubation was used for the comparative analysis.

To investigate whether the enhancement of muraymycin production corresponds to that of the transcription level of the *mur* genes in DM-5 mutant, gene transcription analysis was performed based on the transcriptional unit of the *mur* genes. Bioinformatic analysis indicated that *mur12*-*mur31* constitutes an operon. For this reason, the transcription of the intergenic region between genes was initially detected to identify the polycistrons, and results (Fig. 3A) showed that the lack of positive PCR signals between *mur10* and *mur11*, *mur33* and *mur34*, *mur34* and *mur35*, and *mur36* and *mur37*, proposing that these genes were located in independent transcriptional units. Moreover, the positive PCR signals, as predicated, indicated that the co-transcription of genes from *mur12* to *mur31*, and *mur32* and *mur33* share another transcriptional unit.

To comparatively analyze the transcriptional level of *mur* genes in DM-5 by real time PCR [26]-[27], five genes, *mur11*, *mur12*, *mur27*, *mur28* and *mur33* were randomly selected for their different location and predicted different functions, and both of DM-5 and WT strains were grown for 96 hr on account of the most dramatic transcription difference (Fig. 3B), results showed that all genes chosen have dramatic increase of transcription level in DM-5 compared with that in the wild type strain (Fig. 3C). Apparently, the increase of transcription level for *mur12*, *mur27* and *mur28* are similar except that of *mur33* due to their divergent promoters. All these suggest that the transcriptional level of *mur* genes completely corresponds to the enhancement of muraymycin production in DM-5 mutant.

As the one promoter between *mur11* and *mur12* initiates the transcription of genes from *mur12* to *mur31*, another middle gene *mur17* was randomly selected for *mur33* mutant strain (DM-6) to detect the transcription difference. Results (Fig. S4, C) showed that the transcription of *mur17* in the wild type strain is prominently higher than that of DM-6 (700-fold at 72 hr, 50-fold at 96 hr, and 2-fold at 120 hr), demonstrating the probable positive role of *mur33* in muraymycin biosynthetic pathway.

### Interaction of Mur34 with the target promoter

**Figure 4:**
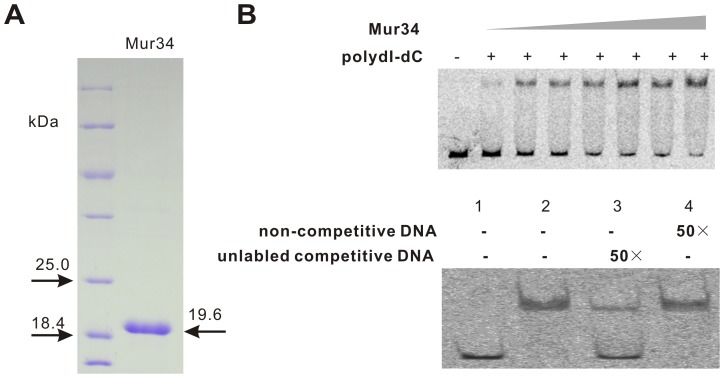
EMSA analysis of His_6_Mur34. (A) SDS-PAGE analysis of His_6_Mur34, the theoretical molecular mass of His_6_Mur34 is 19.6 kDa. The Mur34 protein was loaded into 12% SDS-PAGE for analysis. (B) EMSA analysis of Mur34 and *mur33* promoter. For the above figure, 50-fold of poly dI-dC was added to the each reaction system with an increasing amount of Mur34. For the competitive assay (below), lower case of all samples contain 2.6×10^−4^ M promoter DNA of *mur33* (90-bp specific DNA), for samples 2, 3 and 4, extra 9×10^−6^ M His_6_Mur34 was individually contained. Moreover, 50-fold of unlabelled competitive DNA was added to the reaction system (band 3), and 50-fold of unspecific non-competitive DNA to the system (band 4). Band designations, 1, free DNA; 2-4, protein-DNA complexes.

In order to further characterize Mur34, the gene was cloned into pET28a and expressed in *E. coli* BL21(DE3). As assessed by SDS-PAGE, the His_6_Mur34 exhibits a molecular mass of 19.6 kDa, conforming to the theoretical molecular weight of His_6_Mur34 (Fig. 4A).

To fully elucidate the regulatory mechanism of Mur34, the interaction of Mur34 with target promoters was analyzed. P-mur34, P-mur33, P-mur10, P-mur11/12 and P-mur36/37 were PCR products amplified from the promoter regions of *mur34*, *mur33*, *mur10*, *mur12* and *mur36* by their corresponding primers (Table S2 in File S1). EMSA results showed that P-mur33 was shifted in all of the selected promoters (Fig. S5, band 4-7), and P-mur36/37 (Fig. S5, band 7) possessed a slight shift only in a higher protein concentration compared with that of the others. Additional EMSAs results indicated that Mur34 could not have a binding activity with P-mur36/37 (data not shown). Moreover, it was showed that P-mur34 could not be shifted at all, suggesting that Mur34 could not bind to its own promoter region. In order to preliminarily narrow down the essential binding region of P-mur33 for further identification, the detected *mur33* promoter regions was truncated to several overlapping fragments for further EMSA assays, and results indicated that a length of 90-bp DNA fragment was found to be essential for the binding with His_6_Mur34 (data not shown). Further competitive EMSA results showed that most of the labeled specific DNA fragment could be easily competed by 50-fold concentration of unlabeled one (Fig. 4B, band 3), however, the 50-fold concentration of unspecific DNA was not capable of competing to bind to Mur34. The results suggest that the binding of Mur34 with *mur33* promoter is specific.

### Analysis of the mur33 promoter via catechol dioxygenase activity assay

**Figure 5:**
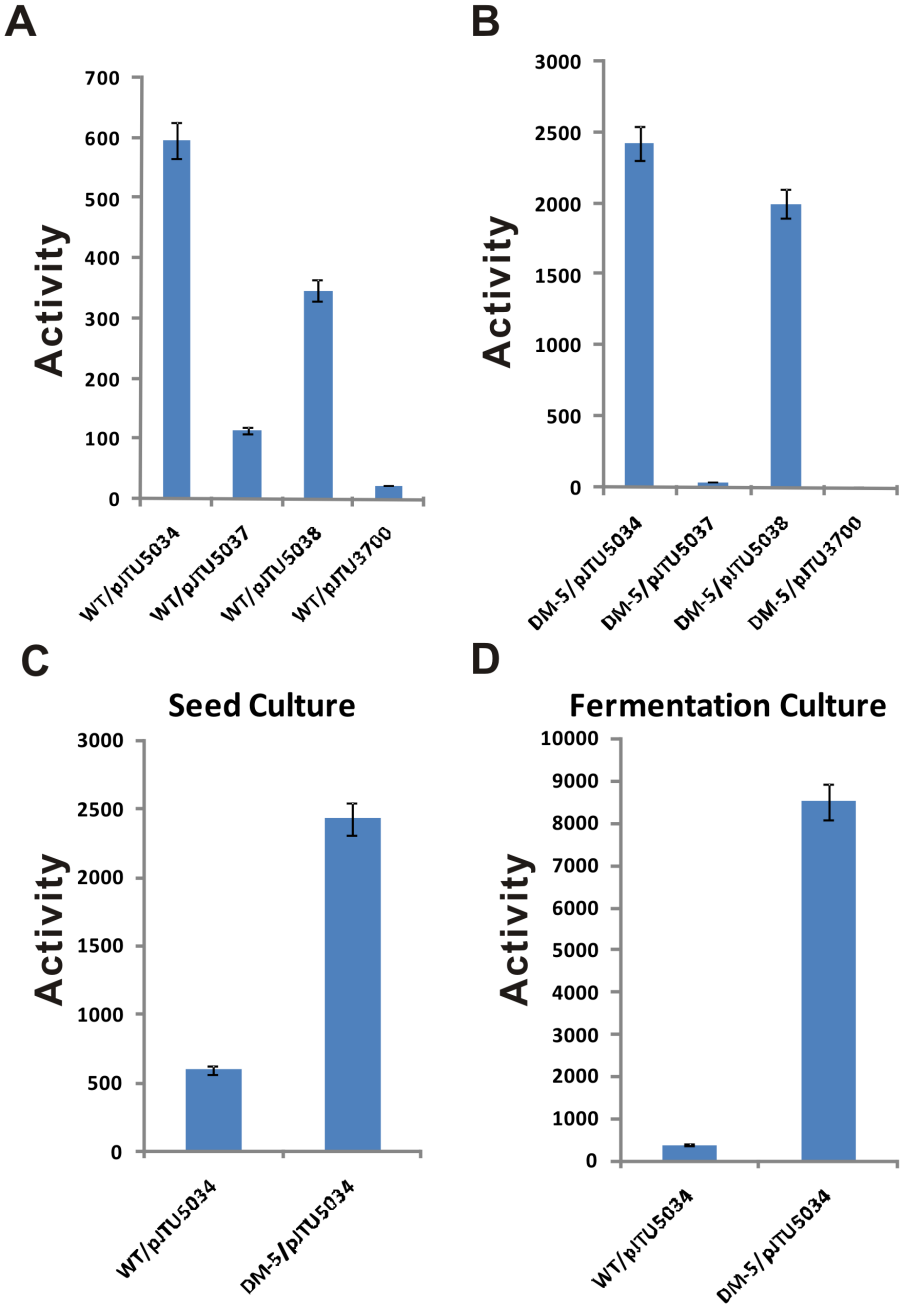
Analysis of *mur33* promoter by catechol dioxygenase activity assay. (A) The enzyme activities for the seed cultures of WT/pJTU5034, WT/pJTU5037 and WT/pJTU5038. (B) The enzyme activities for the seed cultures of DM-5/pJTU5034, DM-5/pJTU5037 and DM-5/pJTU5038. (C) The enzyme activities for the seed cultures of WT/pJTU5034 and DM-5/pJTU5034. (D) The enzyme activities for the fermentation cultures of WT/pJTU5034 and DM-5/pJTU5034. All histograms showed the quantitative catechol dioxygenase activity of *Streptomyces* sp. NRRL30471 and DM-5 independently containing pJTU5034, pJTU5037, pJTU5038 and pJTU3700. WT/pJTU3700 indicates *Streptomyces* sp. NRRL 30471 containing pJTU3700 (no *mur33* promoter) is as the negative control. WT/pJTU5034, indicates *Streptomyces* sp. NRRL 30471 containing pJTU5034 (natural *mur33* promoter). WT/pJTU5037 indicates *Streptomyces* sp. NRRL 30471 containing pJTU5037 (the -10 region mutated on *mur33* promoter). WT/pJTU5038 indicates *Streptomyces* sp. NRRL 30471 containing pJTU5038 (the -35 region mutated on *mur33* promoter). Likewise, DM-5 derived strains were designated.

**Figure 6:**
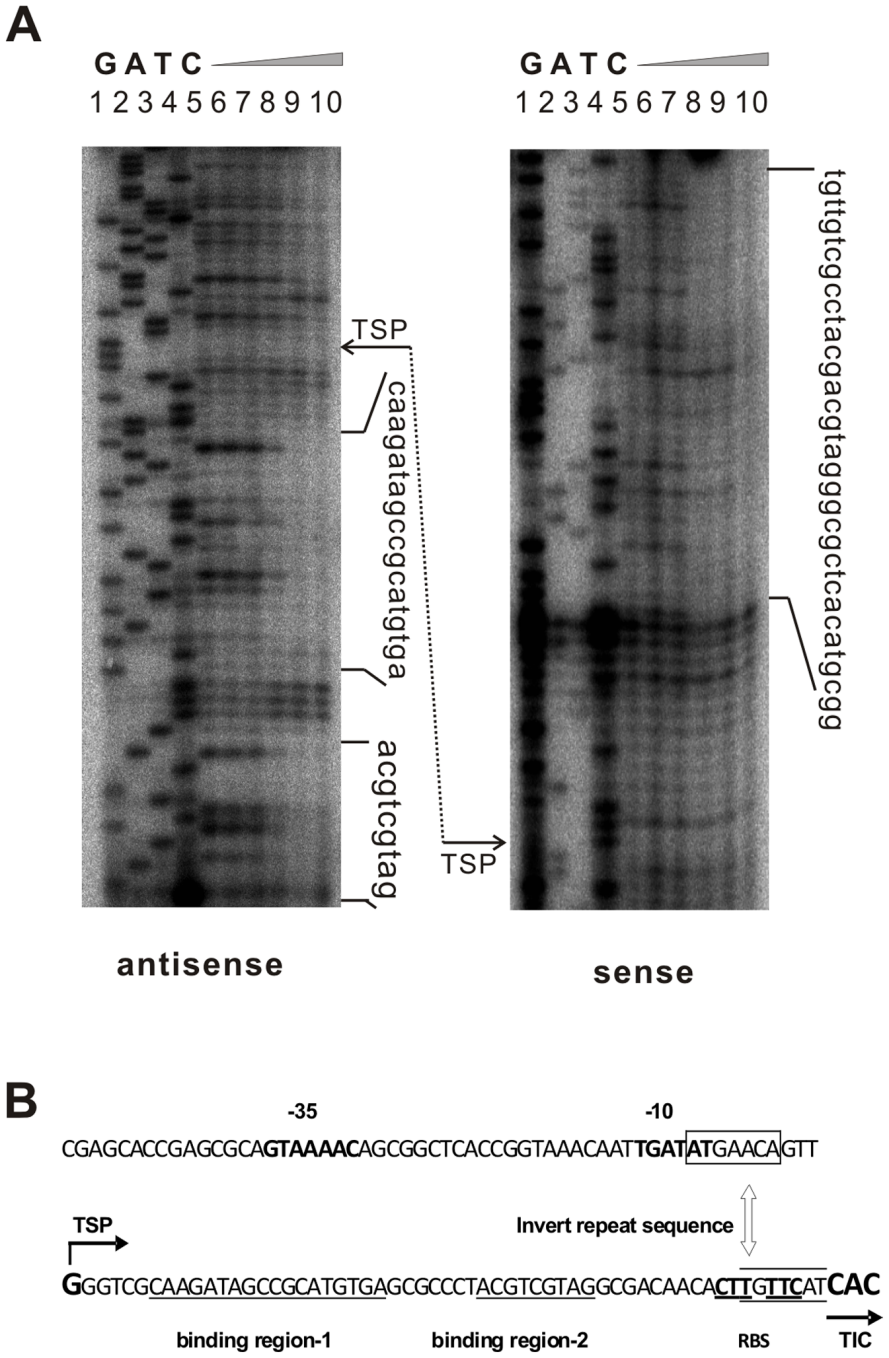
Analysis of the Mur34 binding site by DNase I footprinting assay. (A) Analysis of antisense strand γ-^32^P labeled DNA (left) and the sense strand γ-^32^P labeled DNA (right) upstream of *mur33*. Lanes G (1), A (2), T (3) and C (4) are sequencing ladder. Samples from lands 5–10 contain the same amount of the binding DNA with an increasing amount (0–3.2 µg µl^-1^) of purified His_6_Mur34. The complexes from the samples were digested by DNase I (0.004U per10 µl) at 30°C for 1 min. The vertical sequences to the right of each gel picture indicate the DNA regions protected from the cleavage of DNase I. The transcription start point (TSP) was shown for each DNA strand. (B) “G” indicates the TSP. The sequences underlined were the protected regions by His_6_Mur34 under DNase I, “CAC” indicates the translation initiation codon (TIC), the bold regions upstream of TSP are -10 “TGATAT” and -35 “GTAAAACAG” regions. The bases in the boxes found are palindromes, and the bold and underlined bases near the TIC are supposed to be the Shine-Dalgarno consensus.

To predict the -10 and -35 region and determine the precise binding site of Mur34, the TSP of *mur33* was necessarily identified by 5′ rapid-amplification of cDNA ends [28]. “G” represents the TSP of *mur33* as shown in Fig. 6B, therein deducing the -10 (TGATAT) and -35 (GTAAAAC) regions on *mur33* promoter, the two regions were also analyzed based on the consensus region of the major and essential sigma factors [29]. The *xylE* of *Pseudomonas putida* coding for a catechol 2, 3-dioxygenase that converts a colorless catechol to intensely-yellow 2-hydroxymuconic semialdehyde [30], was used as a reporter gene to evaluate the activities of natural promoter of *mur*33. Results showed that the amount of the reaction product was distinct in different strains, the promoters in WT/pJTU5034 (Fig. 5A) and DM-5/pJTU5034 (Fig. 5B) display the most potent activity, proving that the natural promoter keeps active at both of the incubation stages of *Streptomyces* sp. NRRL 30471. Furthermore, the activity of XylE conspicuously decreased in the -10 mutation strain cultures, WT/pJTU5037 (Fig. 5A) and DM-5/pJTU5037 (Fig. 5B), probably demonstrating the necessity of this region for the binding of RNA polymerase complex. Nevertheless, the -35 region plays an unessential role as indicated in WT/pJTU5038 (Fig. 5A) and DM-5/pJTU5038 (Fig. 5B). In order to find if Mur34 play a negative role in the process, the XylE activity in the cultures of the wild-type and the *mur*34 mutants were detected and compared, and results showed that the natural promoter of *mur*33 in DM-5/pJTU5034 exhibited much more activity than that of WT/pJTU5034 whether the strain growing on the conditions of the seed or fermentative mediums (Fig. 5C, D).

### The regulation mode of Mur34

For further understanding the regulation mode of Mur34, the TSP was identified by 5′RACE, and results indicated that the *ca*. 90-bp specific DNA locates between +60 and −29 relative to the TSP (Fig. 6B). To accurately pinpoint the minimally essential binding region, the complex of *mur33* promoter with Mur34 was treated by DNase I, and results (Fig. 6A) showed that two regions about 28-bp, “CAAGATAGCCGCATGTGA” and “ACGTCGTAG”, were protected by His_6_Mur34 to avoid the cleavage of DNase I at the antisense strand, and a longer region of “TGTTGTCGCCTACGACGTAGGGCGCTCACATGCGG” was protected on the other sense strand. The binding sites exhibit inconsistency to the conservative binding FARE boxes of FarA, which acts as a repressor in the regulation of nucleoside antibiotics [31]. Although differences exist between the two single-stranded DNA, the binding sites both locate at the downstream of TSP of *mur33*. Results suggested that Mur34 repress the transcription unit of the *mur32–33* by preventing the access of RNA polymerase complex.

## Discussion

The nucleoside antibiotic muraymycin was well documented as promising lead compound with clinic potentials since its discovery [5], [32]. The chemical structure and potent bioactivity of muraymycin are known, but the precise regulatory mechanism for its biosynthesis remains obscure. Indeed, the identification and characterization of Mur34 as the novel negative regulator involved in muraymycin biosynthesis, not only provides insights into the regulatory mechanism of the homologous proteins, but may also sets a paradigm for the understanding of a universal regulatory strategy involved in secondary metabolites biosynthesis.

Mur34 shows high homology to a large number of uncharacterized proteins including LviI (74% identity, Lividomycin), RacA (77% identity, Robostamycin), KanI (74% identity, Kanamycin) and NeoR (74% identity, Neomycin). Apparently, this family of proteins is diversely distributed and probably plays a key role in nature, particularly, in microbial secondary metabolites biosynthetic pathways, however, little is known for the precise function of this family of proteins. In the present paper, Mur34 was identified as a regulator that represses the biosynthesis of muraymycin by binding to the promoter region of *mur33–32*. Mur33 shows 61% identity to SsaA (AGG82463.1), which is more recently confirmed to be a novel positive regulator involved in the biosynthesis of sansanmycins [33]. Mur33 is also highly homologous to other proteins including NpsM (ADY76675.1, 60% identity) and PacA (ADN26237.1, 61% identity) in the individual biosynthetic pathways of napsamycin [34] and pacidamycin [35]. All of these proteins harbor a homologous DNA-binding domain in the C-terminal (Fig. S1, B). Although tried with great pains, we could not obtain any soluble protein for further EMAS analysis. However, mutation of *mur33* on the chromosome DNA significantly resulted in large decreases of the inhibition zone and production of antibiotics (Fig. S4, B). In additon, the transcription level of the muraymycin gene cluster also decreased as indicated by the transcription of *mur17* at different growth stages (Fig. S4, C). From these aspects, Mur33 probably play a positive role in the regulation of muraymycin biosynthesis. As a result, we proposed that Mur34 performs the negative regulation of muraymycin production via indirect process by negative controlling of the transcription of *mur33*, which is a positive regulatory gene as established by genetic analysis.

Mur32 belongs to metallophosphatase (MPP) superfamily as analyzed by SMART, and shares a conservative domain with the active site usually composed of metal ions manganese coordinated with octahedral geometry by a cage of histidine, aspartate, and asparagine residues. The individual *mur32* mutant (Fig. S6, A) was also found to be capable of producing muraymycins (Fig. S6, B). Of course, the nearly-unaffected production of muraymycin indicates the existing of alternative gene(s) in other place(s) of the genome.

It is true that Mur34 ascends the pyramid top of regulatory network for muraymycin biosynthetic pathway and directly binds to the region upstream of *mur33*. Mur34 family regulators were distinguished for their unusually-small molecular weight, and the arising curiosity is that how does the Mur34 act to carry out its function. The existed differences of the binding sites between the two DNA strands implicate that both DNA strands probably act with Mur34 in an unknown mode. Simultaneously, we found that the 16-bp region “ACTACTTGTTCACAAC” in the vicinity of the initial codon of *mur33* plays an important role in the binding of Mur34 (Fig. S7), while the detailed reason is unknown. Moreover, the 7-bp invert repeats “ATGAACA” was identified in the downstream of the binding areas (Fig. 6B), and whose function is likely to help the blocking the access of RNA polymerase complex. Therefore, it would be most interesting to elucidate the dynamic repression mechanism of Mur34 with the binding regions, and the related research now waits to be further investigated.

In summary, we report the identification and characterization of Mur34 as a novel negative regulator involved in muraymycin biosynthesis, and disruption of *mur34* contributes to a significantly enhanced muraymycin production. We detected the target binding areas of Mur34 at the downstream of TSP and also found a 16-bp region on the promoter region of *mur33* that may play an important role for the Mur34 binding process. We anticipate that understanding of the precise regulation mechanism and the regulative network for the Mur34 family proteins in *Streptomyces* would be of great potential for rational improvement of secondary metabolite productions.

## Materials and Methods

### Bacterial strains and plasmids, primers and sequencing, growth conditions and useful kit

Strains, plasmids and primers used are listed in Table S1 and Table S2 in File S1, respectively. Primers were synthesized at Shanghai Sangon Biotech (China). PCR was performed using rTaq (TaKaRa), KOD plus (Toyobo) and Pfu DNA polymerase (NEB), and DNA sequencing was performed by Invitrogen or BioSun Biotech (China).

*Escherichia coli* was grown in Luria-Bertani (LB) at 37°C or 30°C (Protein overexpression), *Streptomyces* strains were grown in TSBY or on ISP_4_ (Oxid) agar medium supplemented with proper antibiotics at 30°C (Table S3 in File S1). *Bacillus subtilis* was used as indicator strain for bioassay. MS agar plate was used for *E. coli*-*Streptomyces* conjugation.

*E. coli* plasmids were prepared by alkaline lysis [36]. *Streptomyces* cultures used for RNA extraction were grown in fermentative medium (BPM21, detailed recipe in File S1) [2]. Total RNA isolations were carried out according to methods by Hopwood *et al* [24] with RNA isolation kit (SBS, China). Reverse transcription (the RevertAid™ H Minus First Strand cDNA Synthesis Kit) and real-time PCR kit were from Fermentas, 5′RACE was done according to the user bulletin (Invitrogen). The DNase I footprinting Sequencing kit was purchased from USB (US).

### Construction, complementation and identification of related mutants

For construction of DM-5 and DM-7 mutants, a 14.6-kb EcoRV digested DNA fragment including the target gene from cosmid 18F3 was cloned to pBluescript II SK(+) to give pJTU5629, then the EcoRV-SpeI engineered 14.6-kb fragment from the resulting plasmid was cloned into the counterpart sites of pOJ446 [37] to produce pJTU5633. The *neo* targeting cassettes were obtained from SupercosI with primers mur34F/mur34R and mur32F/mur32R by PCR, after that, the two purified fragments were used to recombine into *mur34* and *mur32* by PCR-targeting [25], individually generating pJTU5634 and pJTU5039, which were confirmed by PCR with corresponding primers (mur34-det-F/mur34-det-R, mur32-det-F/mur32-det-R). The *mur34*::*neo* (DM-5) and *mur32*::*neo* (DM-7) mutants were screened and validated by PCR. To generate in-frame deletion vector of *mur33*, the 1146-bp XbaI/XhoI engineered left arm and 1355-bp XhoI/SpeI engineered right arm were simultaneously cloned into the related sites of pOJ446 to give rise to pJTU5020. After confirmed by PCR using primers mur33-det-F/mur33-det-R, the plasmid was conjugated into *Streptomyces* sp. NRRL30471 for the construction of *mur33* in-frame deletion mutant on the basis of the standard methods [24]. A 704-bp DNA fragment containing the promoter and the coding regions was amplified using mur34-selfF and mur34-selfR (Table S2 in File S1) as primers, then cloned into the BamHI and EcoRV of pSET152. The resulting expression vector pJTU5052 was introduced into DM-5 by conjugation to give the complementary strain DM-14. The mutations of *mur34* on 18F3, mutations of *mur11* and *mur12* on pJTU5030 were all constructed by PCR-Targeting, the kanamycin resistance *neo* cassette obtained by using primers of mur34F/mur34R, mur11F/mur11R and mur12F/mur12R separately (Table S2 in File S1). The obtained disrupted mutations of *mur34*, *mur11* and *mur12* were confirmed by using primers of mur34-det-F/mur34-det-R, mur11-det-F/mur11-det-R and mur12-det-F/mur12-det-R (Table S2 in File S1). The inframe-deleted mutations of *mur34* and *mur12* were made by using *in vitro* XbaI and SpeI digestion (unique sites) and relegation, and confirmed by PCR.

### Production, purification and detection of the muraymycins

For production of muraymycins, the spores of *Streptomyces* sp. NRRL 30471 or its derivatives were inoculated into TSBY media and incubated at 220 rpm for 2 days, then 2% of the pre-cultures were inoculated into the fermentative medium for additional 5 days incubation. For rapid purification of muraymycins, the cultures were pretreated and concentrated by WCX cation column (Waters) (The detailed procedure in File S1). 10 µl of the concentrated extract was submitted for bioassay and LC-MS analysis. LC-MS analysis was performed on Agilent HPLC system equipped with a SB-C18 (3.5 µm, 4.6×250 mm) column (Agilent) coupled to the 1100 Series LC/MSD Trap mass spectrometer (The detailed methods used for the detection of the muryamycins in File S1). The flow rate was 0.3 ml min^−1^ and muraymycins were detected at a UV absorbance of 262 nm. The ion trap mass spectrometer analysis for muraymycins was operated in positive mode. The parameters for all MS analysis are as follows: drying gas flow was 10 litres min^−1^, nebulizer pressure was 30 psi and the drying gas temperature was 350°C.

### PCR and real-time PCR

The PCR reaction system and conditions used are the same except the special annealing temperature for each target gene. Quantification and purity analysis of all PCR products were detected by using NanoDrop ND-1000 Spectrophotometer (Thermo Scientific) and DNA agarose gel electrophoresis. Gene expression analysis by reverse transcription and real-time PCR was performed as previously described [27], [38]–[39].

For isolation of total RNA, 1 ml of fermentation cultures of four independent isolations at individual 48, 72, 96 and 120 h incubation time were detected for DM-5 and the wild-type strains, and DM-6. The concentration, purity and integrity of the RNA samples were evaluated by spectrophotometry and agarose gel electrophoreisis. Total RNA samples were digested by RNase-free DNase I at 37°C for about 4 h, and the quality of DNA-free RNA was detected by amplification of the 16s rDNA with primers 16-F/16-R. After that, the DNA-free RNAs were reversely transcribed for the detection of intergenic region on muraymycin gene cluster and further for real-time PCR analysis.

For real time PCR detection, the reactions were performed on an ABI7500 Fast Real Time System (Applied Biosystems), and each reaction (20 µl) system contained 0.5–250 ng of cDNA depending on dilution, 10 µl of SYBR Green qPCR Master Mix (Fermentas), 50 pM of both of the forward and reverse primers. The sizes of PCR products are 122-bp for *mur11*, 111-bp for *mur12*, 126-bp for *mur27*, 112-bp for *mur28*, 118-bp for *mur33*, and 109-bp for *mur17*. The conditions for PCR reactions were 95°C for 10 min followed by 40 cycles of 95°C for 15 s, 60°C for 1 min. The transcription difference of the *mur34* mutant corresponding to wild type strain (DM-5/WT), and transcription difference of the wild type strain corresponding to *mur33* mutant strain (WT/DM-6) were obtained using the detailed methods in File S1.

### 5′RACE and catechol dioxygenenase activity assays

For 5′ RACE experiment, the first strand cDNA synthesis is primed using the gene-specific antisence oligonucleotide GSP1-1,then the first strand product is purified from unincorporated dNTPs and GSP1-1. After cDNA was dC-tailed, a primer mixture (a nested gene-specific primer GSP2-1, a combination of a complementory homopolymer-containing anchor primer, and corresponding adapter primer AAP) were allowed to amplify the unknown sequences between the GSP2-1 and 5′-end of the mRNA. The replica obtained was amplified using AUAP and nested GSP3-1, eventually, the proper amplicon was ligated to pMD18-T vector for sequencing.

For the construction of engineered strains for catechol dioxygenenase activity assay, a 717-bp promoter sequence from +351-bp to -365-bp of *mur33* was cloned by mur33-P-PF/mur33-P-PR, and sequenced, then the BamHI-SacII engineered DNA fragment was inserted into pJTU3700 forming pJTU5034, which was further conjugated into DM-5 and the wild-type strain to generate DM-5/pJTU5034 and WT/pJTU5034. The conjugants were confirmed by PCR using the general primers M13F/M13R. -10 and -35 regions of *mur33* promoter were mutated using 5′-phosphorylated primers of P33-10F/P33-10R and P33-35F/P33-35R, and the mutated templates were independently cloned into pJTU3700 to form pJTU5037 and pJTU5038. The constructs were subsequently conjugated into the *Streptomyces* sp. NRRL30471 forming WT/pJTU5037 and WT/pJTU5038, and into DM-5 forming DM-5/pJTU5037 and DM-5/pJTU5038 as confirmed by PCR. Similarly, pJTU3700 was integrated into the chromosomal DNA of DM-5 and wild type strain, forming DM-5/pJTU3700 and WT/pJTU3700 as negative controls.

For catechol dioxygenenase activity assay, 5 ml broth was harvested and washed in 20 mM potassium phosphate with pH7.2. After that, the cells were suspended in 3 ml sample buffer. The crude proteins were obtained after cell lysis by sonication and cells debris was wiped out at 4°C [40]. A quantity of cell-free extract was adjusted to produce a linear change over the time for measurement. The reaction was initiated by addition of cell-free extracts, and the data was recorded per minute in total time of 10 min at the absorption of 375 nm. Catechol dioxygenase activity was calculated according to the formerly described methods [41]. Protein concentrations were determined by a method of Bradford [42].

### Overexpression and purification of Mur34

*mur34* amplified by using KOD Plus polymerase with primers mur34-det-F/mur34-det-R was treated by NdeI and BamHI, then it was cloned into the corresponding sites of pET28a to generate pJTU5036. After confirmation, the expression construct was transformed into *E. coli* BL21(DE3) [43]. For overexpression of Mur34, 1% of the overnight culture of *E. coli* BL21(DE3)/pLysE/pJTU5036 was inoculated into a fresh LB medium with proper antibiotics. After growing to OD_600_ = 0.4 at 37°C, isopropyl β-D-1-thiogalactopyranoside was added to broth with a final concentration of 0.4 mM, then the culture was incubated for another 4 h at 30°C. Cells were harvested and resuspended in A buffer and lysed by sonication in ice bath. After centrifugation at 4°C, the supernatant was loaded into HisTrap HP column (GE Healthcare), and then the target protein was eluted of a linear gradient 0-500 mM with imidazole by AKTA Purifier with Frac-900 (GE Healthcare). After that, the protein was desalted by a HisTrap Desalting column (GE Healthcare) and stored in protein stock buffer with 10% glycerol at −80°C. The purified His_6_Mur34 was measured by 12% SDS-PAGE analysis, and the protein concentration was determined by the Bradford method.

### Electrophoretic mobility shift and DNase I footprinting assays

For EMSA, the purified His_6_Mur34 was used to bind the target promoter regions. Individual strand of the amplicons used in EMSA and DNase I footprinting assay was 5′ end-labeled with γ-^32^P using the following protocol, certain amplicons were incubated with [γ-^32^P]-ATP (Beijing Furui Co.Ltd) using 10 U of T_4_ polynucleotide kinase (Promega) at 37°C for 30 min, followed by 90°C for 2 min to inactivate the enzyme. Five γ-^32^P labeled promoters including P-mur34 (amplified by primers mur34-PF/mur34-PR), P-mur33 (amplified by primers mur33-PF/mur33-PR), P-mur10 (amplified by primers mur10-PF/mur10-PR), P-mur11/12 (amplified by primers mur11/12-PF/mur11/12-PR) and P-mur36/mur37 (amplified by primers mur36-PF/mur36-PR) were mixed together at a relative equivalent concentration. The reaction system including His_6_Mur34 in the binding buffer was incubated at 30°C for 30 min. The samples containing the labeled promoters and His_6_Mur34 with different concentrations were analyzed in a 6% native polyacrylamide gel.

For the competitive EMSAs, the specific promoter regions of *mur33* was amplified by PCR using primers of mur33-PF/mur33-PR11, the promoter DNA was labeled with 5′-FAM synthesized by Sangon Biotech (Shanghai) on the forward primer. 50-fold specific competitor (the same unlabeled specific promoter with the labeled DNA) and 50-fold non-specific competitor (the similar base pair component and length) were separately added into the reaction system, 50-fold poly dI-dC was added into every binding system. After incubation, the DNA-protein complexes and free DNA were separated by 6% native polyacrylamide gels with a running buffer at 4°C. Then the gel was detected by FLA-3000 (FUJI FILM) at 473 nm.

For DNase I footprinting assays, PCR reactions were performed in a total volume of 50 µl containing 2 ng template DNA, 10 pmol of each labled primer, 5% DMSO, and 5U sequencing-grade Taq polymerase (Genescript), and DNA fragment of the shorted P-mur33 were purified using the QIAquick PCR Purification Kit (Qiagen). For binding site analysis, the reaction mixture contained 500 cps ^32^P-lablelled DNA fragments (50 nM), after the binding of protein with DNA, the reaction mixture was incubated in ice bath for 5 min prior to addition of 2.5 µl DNase I buffer and 0.3 U of DNase I (Fermentas), then was carried out for further incubation at 30°C for 1 min. The reaction was stopped by adding of 100 µl stop solution and 50 µl phenol-chloroform. Samples were then denatured at 95°C for 2 min and loaded on 8% polyacrylamide-urea gel for analysis. The DNA sequence ladder was generated using an *fmol* DNA Cycle Sequencing kit (Promega). After electrophoresis, the gels were dried and exposed to a Kodak X-ray film for analysis.

## Supporting Information

Figure S1 ***In silico*** **analysis of Mur34 and Mur33.** (A) Analysis of Mur34 with its homologous proteins was performed by clustal W software. The secondary structure of Mur34 was analyzed by SWISS-MODEL online program (http://swissmodel.expasy.org/). (B) The secondary structure of Mur33 C-terminal was analyzed by SWISS-MODEL, and the homologous proteins from the biosynthetic pathways of translocase I inhibitors were analyzed by clustal W. The red boxes showed the a-helix domains which contribute to the DNA-binding activity.
(PDF)

Figure S2 **Complementation of DM-14 detected by bioassays.** a, b and c indicate the inhibition zones of the metabolites of the wild type strain (*Streptomyces. sp*. NRRL30471), *mur34* mutant (DM-5) and *mur34* complemented strain (DM-14).
(PDF)

Figure S3 **Heterologous expression and determination the minimal muraymycin gene cluster.** (A) For targeted inactivation of *mur34*, a kanamycin resistance cassette (*neo*) from SuperCos1 amplified using the tailed primers mur34F and mur34R (Table S2 in File S1), was recombined into 18F3 by PCR-targeting strategy to give 18F3/*mur34::neo* (pJTU5642). The *neo* cassette was then deleted by XbaI and SpeI digestion (unique sites) and religated to produce 18F3/Δ*mur34* (pJTU5030). The unmarked deletion was confirmed by PCR using primers mur34-det-F and mur34-det-R. (B) MS and bioassay analysis of the metabolites produced by TK24/18F3 and TK24/pJTU5642. (C) Likewise, *mur11* in 18F3/Δ*mur34mur11::neo* (pJTU5024) was inactivated using the primers mur11F and mur11R for *mur11* inactivation in the gene cluster. (D) MS and bioassay analysis of the metabolites produced by TK24/pJTU5024. (E) *mur12* in 18F3/Δ*mur34mur12::neo* was inactivated using the primers mur12F and mur12R, then the *neo* cassette was deleted with the same method to produce 18F3/Δ*mur34*Δ*mur12* (pJTU5053), and mur12-det-F and mur12-det-R were used to confirm the unmarked deletion. The mutants were introduced into a host of *S. lividans* TK24. The metabolites were produced the same as the *Streptomyces sp*. NRRL30471. (F) MS and bioassay analysis of the metabolites produced by TK24/pJTU5053.
(PDF)

Figure S4 **Construction of mur33 mutant, MS and transcription difference analysis.** (A) Representational map for the construction of DM-6 and PCR confirmation. (B) Bioassay and MS analysis of the metabolites, Top, the metabolites produced by the wild type strain. For MS analysis, Muraymycin C1 and D1 components were selected for detection. Bottom, the metabolites produced by DM-6 mutant, the left-up shows the inhibitions zone of the metabolites from each strain. (C) Transcription difference of *mur17* obtained from the relative amount of DM-6 divided by that of the wild type at different fermentation time.
(PDF)

Figure S5 **EMSA analysis of His6Mur34 with promoters on the gene cluster.** Gel retardation of His_6_Mur34 with promoters in muraymycin gene cluster. The numbers show the different reaction, and the obliquely triangular indicates the increasing amount of Mur34. The left characters indicate promoters in the reaction system separated by gel electrophoresis. P-mur10, P-mur11/12, P-mur33, P-mur34 and P-mur36/37 means the promoter fragment PCR-amplified from the region upstream of *mur10*, *mur12* (*mur11*), *mur33*, *mur34* and *mur36* (*mur37*). All samples contained 5.6×10^−5^ M DNA; Samples 2–7 contained extra Mur34 with individual amount of 0.74×10^−6^, 1.85×10^−6^, 3.7×10^−6^, 7.4×10^−6^, 11.1×10^−6^, 22.2×10^−6^ and 44.4×10^−6^ M. The complex of DNA fragment and Mur34 were loaded on 8% wt vol^−1^ polyacrylaminde gel cast with the running buffer. Band designations, 1, free DNA; 2–7, protein-DNA complexes.
(PDF)

Figure S6 **Construction and analysis of** ***mur32*** **mutant.** (A) Representational map for the construction of DM-7 and PCR confirmation. (B) Bioassay and LC-MS analysis of the metabolites produced by DM-7 strain. For LC-MS analysis, Muraymycin C1 and D1 components were selected for detection.
(PDF)

Figure S7 **EMSA analysis of His_6_Mur34 with** ***mur33*** **promoter fragments with different length.** P33-9 and P33-2 are the DNA fragments of *mur33* promoter amplified with primers mur33-PF/mur33-9R and mur33-2F/mur33-9R. The length of the two fragments are different from each other, P33-9 is 16-bp longer than P33-2. The binding complex of Mur34 with promoter DNA was detected by running a 2% agarose gel electrophoresis, stained by EB.
(PDF)

File S1 **The detailed supplemental data including methods, buffers, media and tables.**
(DOCX)
