# Supplementary figures and images for: Identification of Mur34 as the Novel Negative Regulator Responsible for the Biosynthesis of Muraymycin in Streptomyces sp. NRRL30471
Source: PLoS One. 2013 Oct 15;8(10):e76068. doi: 10.1371/journal.pone.0076068 (PMC3797123; doi:10.1371/journal.pone.0076068)

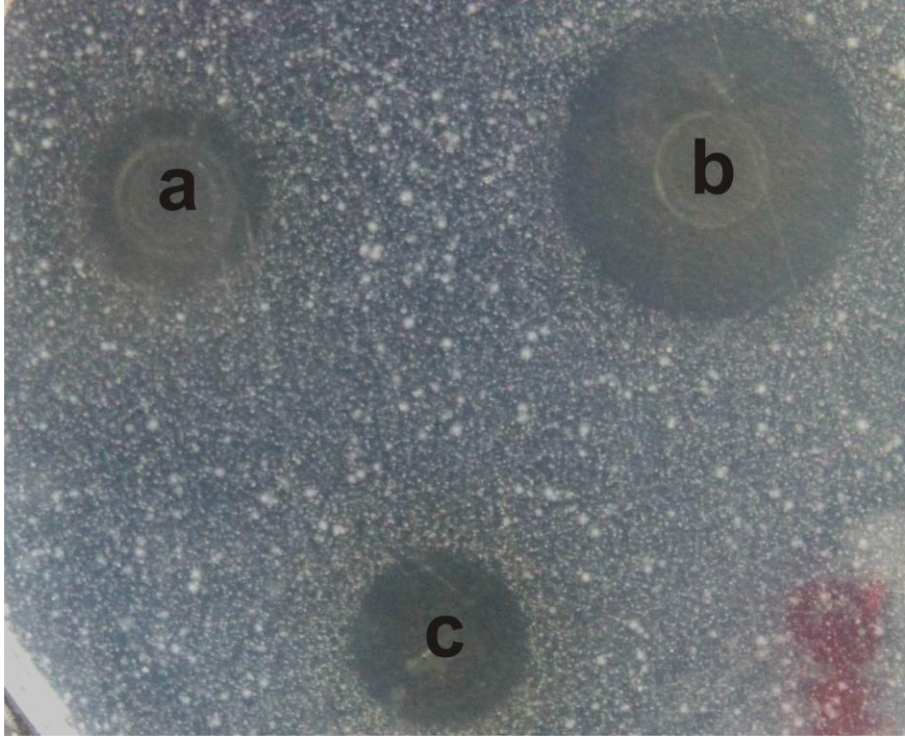

Supplement: Figure S2 — Complementation of DM-14 detected by bioassays. a, b and c indicate the inhibition zones of the metabolites of the wild type strain (Streptomyces. sp. NRRL30471), mur34 mutant (DM-5) and mur34 complemented strain (DM-14). (PDF) [file pone.0076068.s002.pdf]

**A**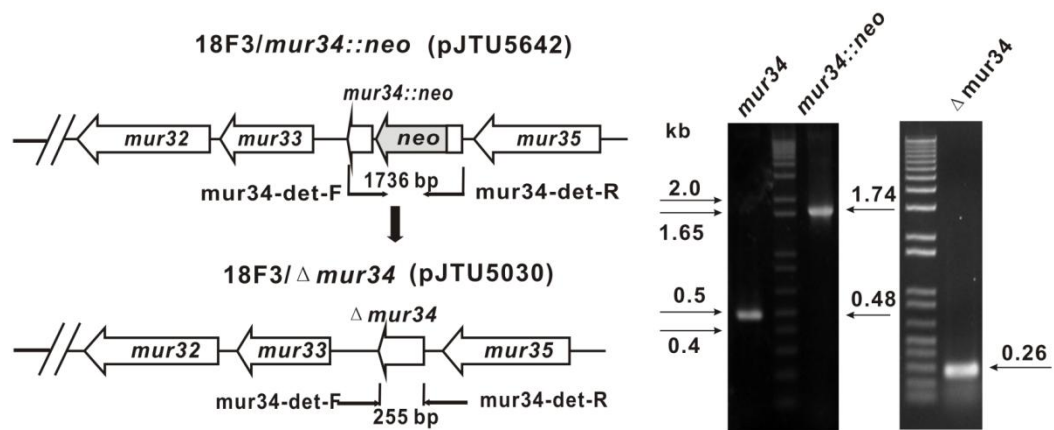**B**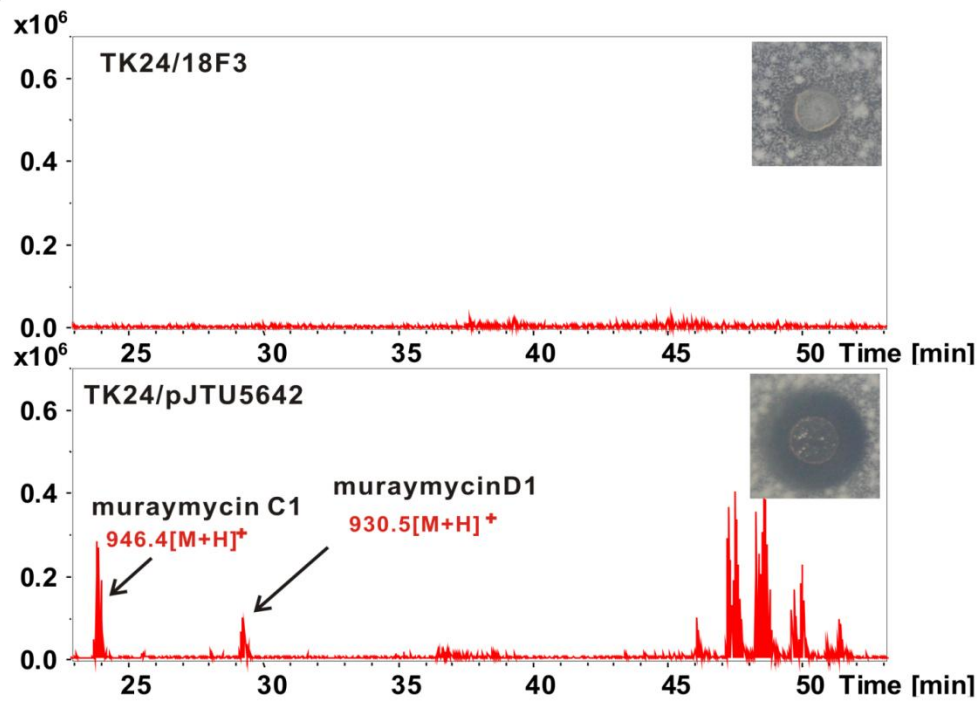

**C**

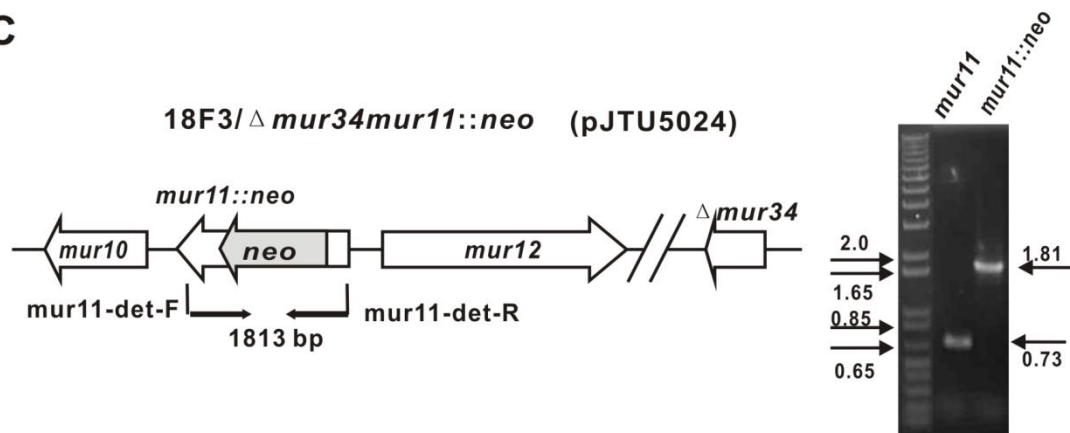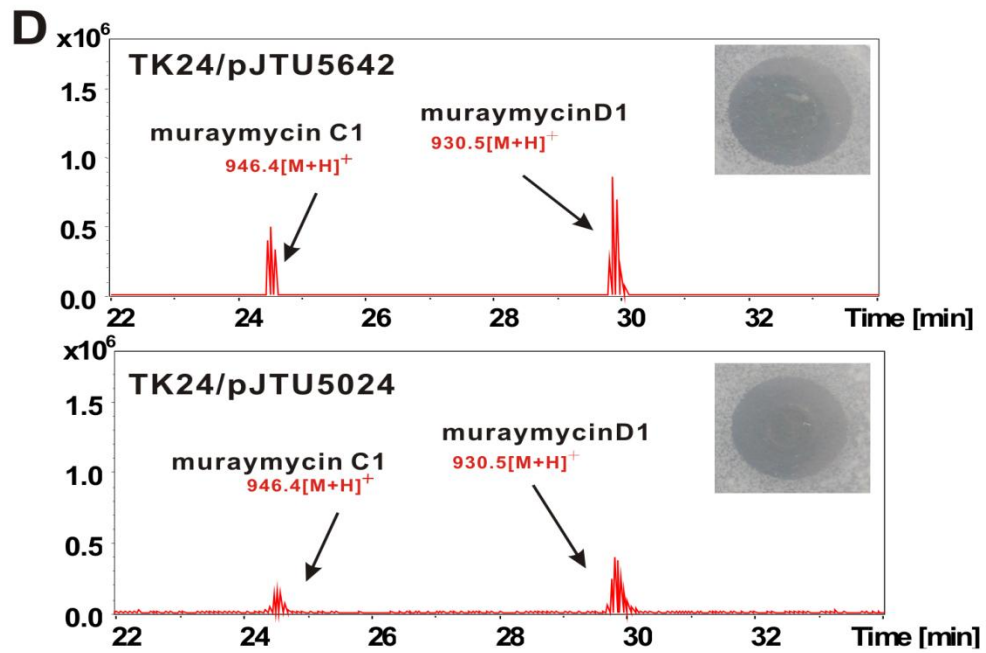

**E**

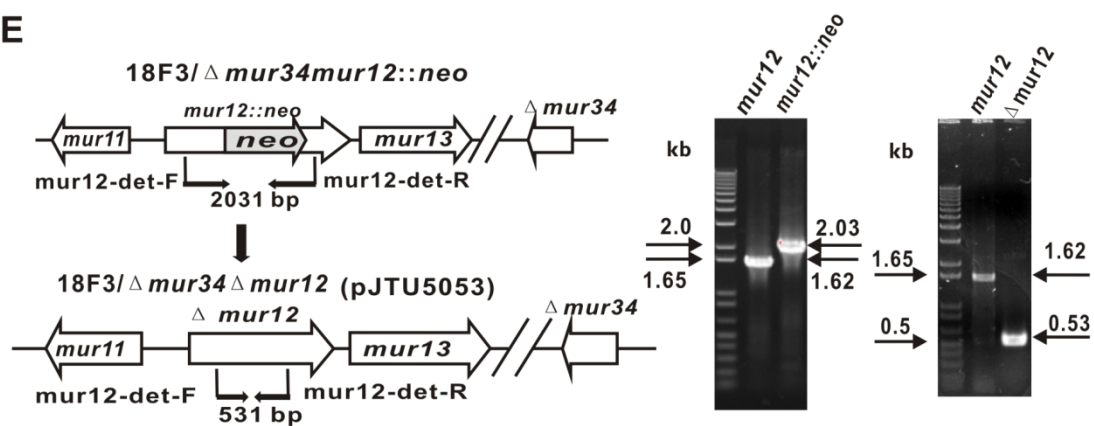

**F**

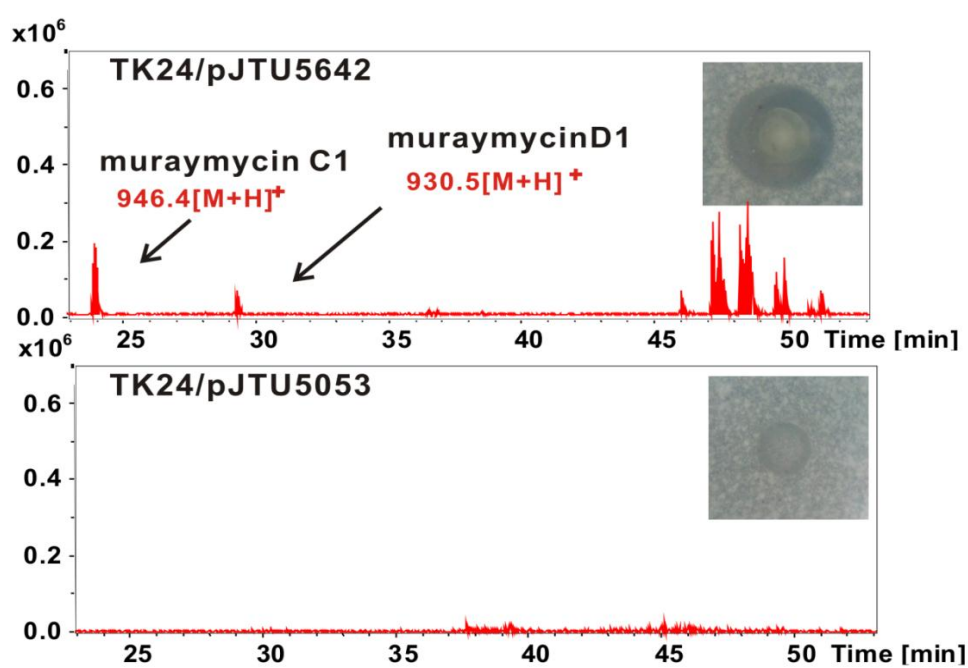

Supplement: Figure S3 — Heterologous expression and determination the minimal muraymycin gene cluster. (A) For targeted inactivation of mur34, a kanamycin resistance cassette (neo) from SuperCos1 amplified using the tailed primers mur34F and mur34R (Table S2 in File S1), was recombined into 18F3 by PCR-targeting strategy to give 18F3/mur34::neo (pJTU5642). The neo cassette was then deleted by XbaI and SpeI digestion (unique sites) and religated to produce 18F3/Δmur34 (pJTU5030). The unmarked deletion was confirmed by PCR using primers mur34-det-F and mur34-det-R. (B) MS and bioassay analysis of the metabolites produced by TK24/18F3 and TK24/pJTU5642. (C) Likewise, mur11 in 18F3/Δmur34mur11::neo (pJTU5024) was inactivated using the primers mur11F and mur11R for mur11 inactivation in the gene cluster. (D) MS and bioassay analysis of the metabolites produced by TK24/pJTU5024. (E) mur12 in 18F3/Δmur34mur12::neo was inactivated using the primers mur12F and mur12R, then the neo cassette was deleted with the same method to produce 18F3/Δmur34Δmur12 (pJTU5053), and mur12-det-F and mur12-det-R were used to confirm the unmarked deletion. The mutants were introduced into a host of S. lividans TK24. The metabolites were produced the same as the Streptomyces sp. NRRL30471. (F) MS and bioassay analysis of the metabolites produced by TK24/pJTU5053. (PDF) [file pone.0076068.s003.pdf]

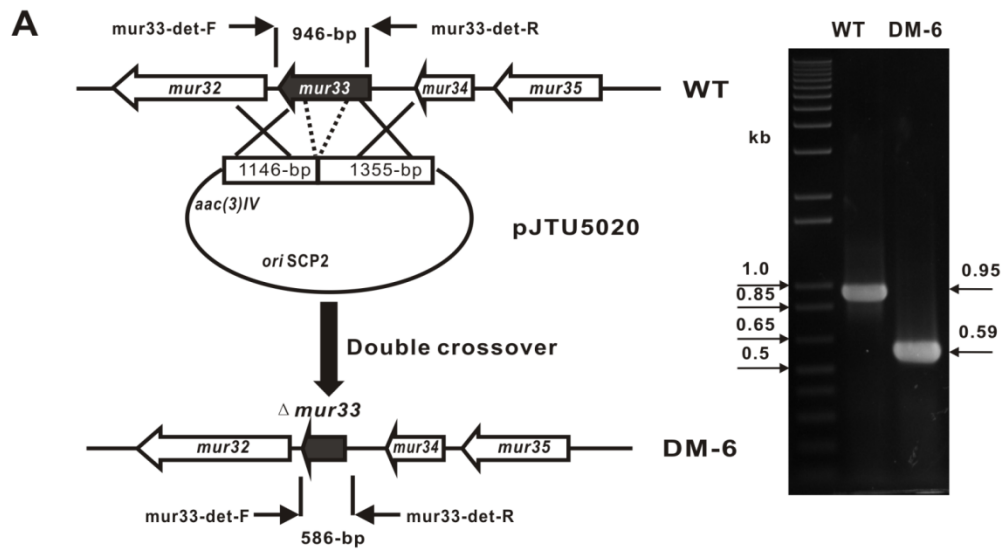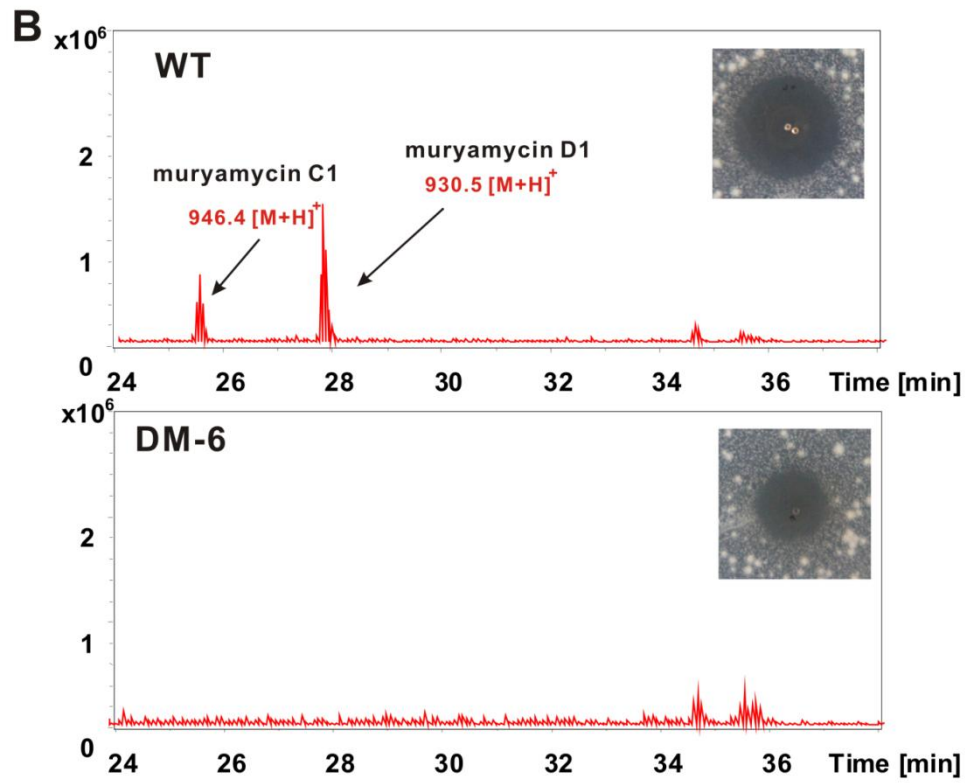

**C**

**Transcription of *mur17* (WT/DM-6)**

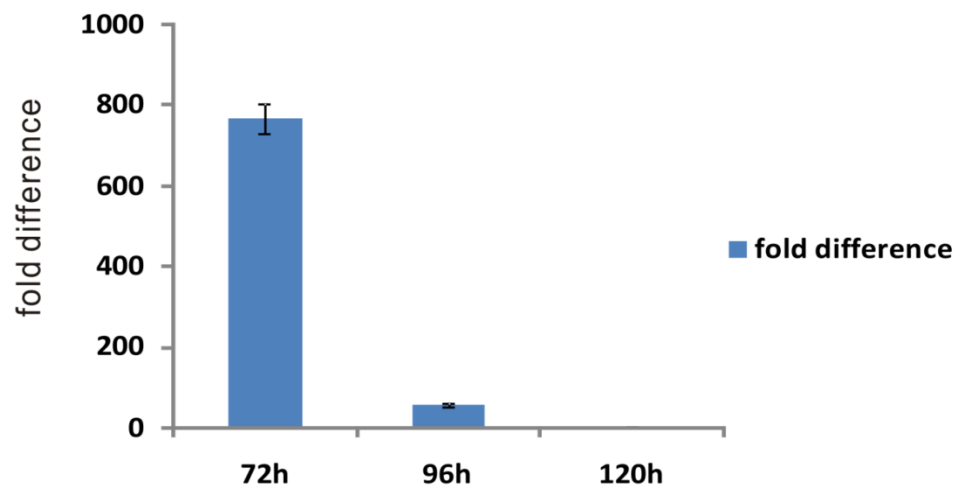

Supplement: Figure S4 — Construction of mur33 mutant, MS and transcription difference analysis. (A) Representational map for the construction of DM-6 and PCR confirmation. (B) Bioassay and MS analysis of the metabolites, Top, the metabolites produced by the wild type strain. For MS analysis, Muraymycin C1 and D1 components were selected for detection. Bottom, the metabolites produced by DM-6 mutant, the left-up shows the inhibitions zone of the metabolites from each strain. (C) Transcription difference of mur17 obtained from the relative amount of DM-6 divided by that of the wild type at different fermentation time. (PDF) [file pone.0076068.s004.pdf]

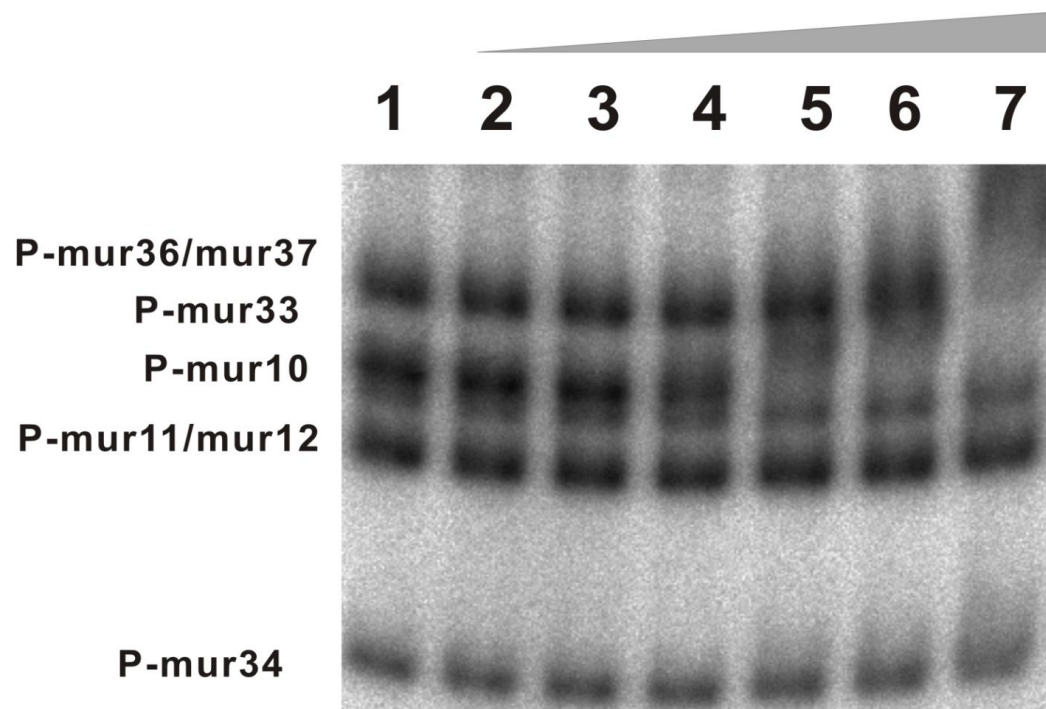

Supplement: Figure S5 — EMSA analysis of His6Mur34 with promoters on the gene cluster. Gel retardation of His6Mur34 with promoters in muraymycin gene cluster. The numbers show the different reaction, and the obliquely triangular indicates the increasing amount of Mur34. The left characters indicate promoters in the reaction system separated by gel electrophoresis. P-mur10, P-mur11/12, P-mur33, P-mur34 and P-mur36/37 means the promoter fragment PCR-amplified from the region upstream of mur10, mur12 (mur11), mur33, mur34 and mur36 (mur37). All samples contained 5.6×10^−5^ M DNA; Samples 2–7 contained extra Mur34 with individual amount of 0.74×10^−6^, 1.85×10^−6^, 3.7×10^−6^, 7.4×10^−6^, 11.1×10−6, 22.2×10−6 and 44.4×10−6 M. The complex of DNA fragment and Mur34 were loaded on 8% wt vol−1 polyacrylaminde gel cast with the running buffer. Band designations, 1, free DNA; 2–7, protein-DNA complexes. (PDF) [file pone.0076068.s005.pdf]

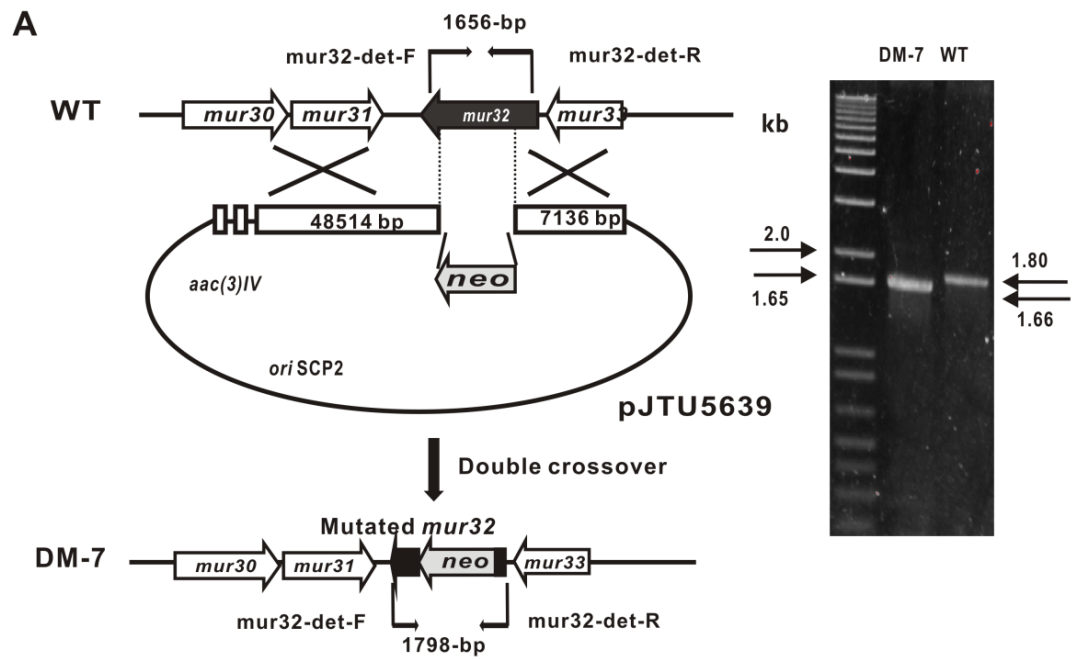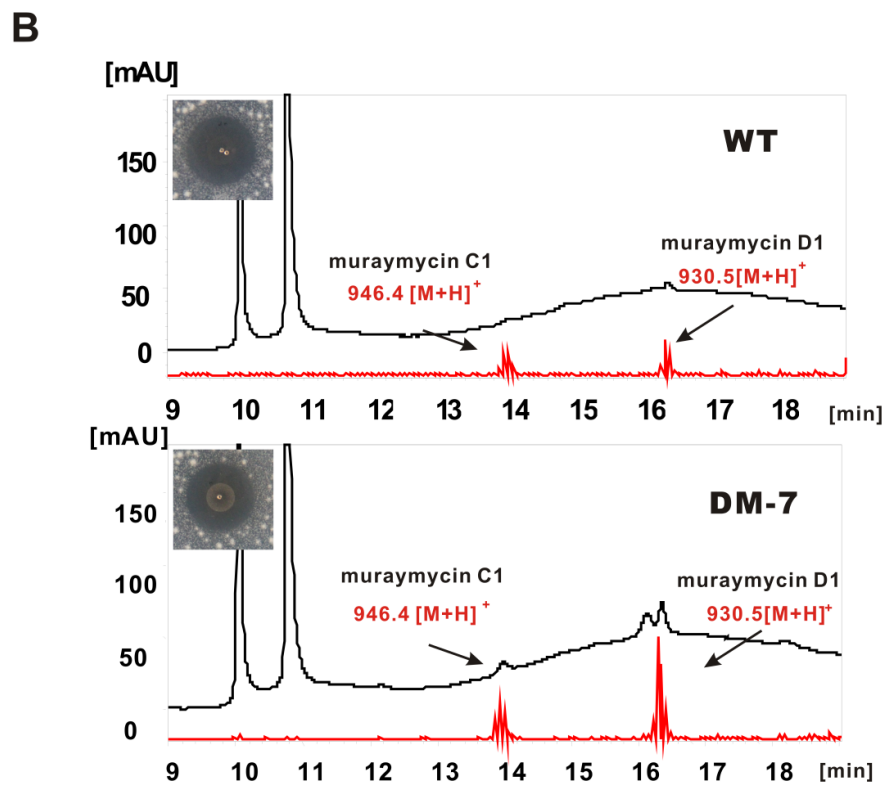

Supplement: Figure S6 — Construction and analysis of mur32 mutant. (A) Representational map for the construction of DM-7 and PCR confirmation. (B) Bioassay and LC-MS analysis of the metabolites produced by DM-7 strain. For LC-MS analysis, Muraymycin C1 and D1 components were selected for detection. (PDF) [file pone.0076068.s006.pdf]

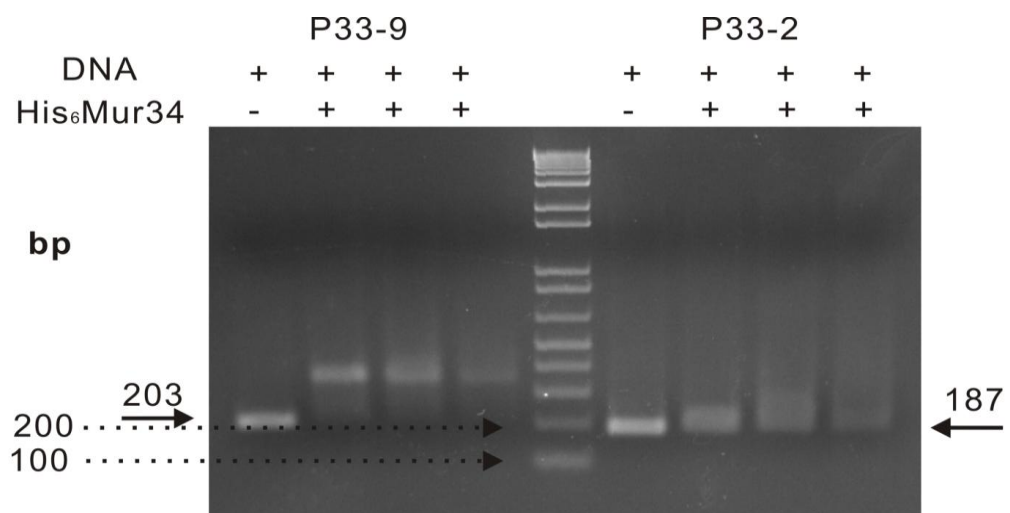

Supplement: Figure S7 — EMSA analysis of His6Mur34 with mur33 promoter fragments with different length. P33-9 and P33-2 are the DNA fragments of mur33 promoter amplified with primers mur33-PF/mur33-9R and mur33-2F/mur33-9R. The length of the two fragments are different from each other, P33-9 is 16-bp longer than P33-2. The binding complex of Mur34 with promoter DNA was detected by running a 2% agarose gel electrophoresis, stained by EB. (PDF) [file pone.0076068.s007.pdf]
