# Supplementary material for: Identification of Mur34 as the Novel Negative Regulator Responsible for the Biosynthesis of Muraymycin in Streptomyces sp. NRRL30471
Source: PLoS One. 2013 Oct 15;8(10):e76068. doi: 10.1371/journal.pone.0076068 (PMC3797123; doi:10.1371/journal.pone.0076068)
Supplement: File S1 — The detailed supplemental data including methods, buffers, media and tables. (DOCX) [file pone.0076068.s008.docx]

# Supplemental Data

# Identification of Mur34 as the Novel Negative Regulator Responsible for the Biosynthesis of Muraymycin in *Streptomyces* sp. NRRL30471

Dongmei Xu^1,3^, Guang Liu^1^, Lin Cheng^2^, Xinhua Lu^3^, Wenqing Chen^1,2^*, Zixin Deng^1,2^*

^1^State Key Laboratory of Microbial Metabolism, and School of Life Sciences & Biotechnology, Shanghai Jiao Tong University, Shanghai 200030, China

^2^Key Laboratory of Combinatorial Biosynthesis and Drug Discovery (Wuhan University), Ministry of Education, and Wuhan University School of Pharmaceutical Sciences, Wuhan 430071, China

^3^NCPC New Drug Research and Development Co., Ltd, North China Pharmaceutical Group Corporation, Shijiazhuang 050015, China

*Address correspondence to:

**Zixin Deng**, State Key Laboratory of Microbial Metabolism, and School of life Sciences & Biotechnology, Shanghai Jiao Tong University, Shanghai 200030, China, Tel: +86-21-62933404; E-mail: [zxdeng@sjtu.edu.cn](mailto:zxdeng@sjtu.edu.cn).

**Wenqing Chen**, Key Laboratory of Combinatorial Biosynthesis and drug Discovery, Ministry of Education, and School of Pharmaceutical Sciences, Wuhan University, Wuhan 430071, China, Tel: +86-27-68756713; E-mail: wenqingchen2010@gmail.com

# Supplemental methods

**Purification of muraymycin from fermentation broth**

The fermentation culture was harvested and acidified with oxalic acid to pH2.5-3.0. After heat-treated at 70℃ for 20 min, the crude extract was centrifuged (3500g, 30 min), and 5 ml supernatant were loaded onto a WCX cation column (Waters) (sequentially prewashed with 10 ml of the following solvent including 100% methanol, 0.5% aqueous trifluoroacetic acid and water), then muraymycins bound to the column were eluted with 5 ml of 70% methanol containing 0.5% trifluoroacetic acid, which was further concentrated in 200 μl of 50% methanol by vacuum pumping.

**LC-MS analysis of muraymycins**

For detection of the muryamycins, a linear gradient from 15% to 30% acetonitrile in aqueous trifluoroacetic acid (0.3%) over 30 min followed by a linear gradient from 30% to 50% acetonitrile in aqueous trifluoroacetic acid (0.3%) over 10 min was used, then a constant concentration of 50% was used to separate the compounds.

**Detection the transcription of intergenic regions of *mur* genes**

All the intergenic region between *mur10* and *mur11*, *mur11* and *mur12*, *mur13* and *mur14, mur15* and *mur16, mur20* and *mur21*, *mur24* and *mur25, mur25* and *mur26, mur26* and *mur27, mur27* and *mur28, mur28* and *mur29, mur29* and *mur30*, *mur30* and *mur31, mur31* and *mur32, mur32* and *mur33*, *mur33* and *mur34*, *mur34* and *mur35*, *mur35* and *mur36*, *mur36* and *mur37* using cDNA as template were PCR amplified. gDNA as a template was used to amplify all the regions as the positive control. DNase-treated RNA templates were used as negative control. The primers used for amplification of the fragments named the orf10/11-F and orf10/11-R, and the similar name of the others. The PCR products were detected on 1.5-2% agarose gel electrophoresis.

**Methods for the analysis of transcription difference**

The amplification efficiency of each gene is probably a little different due to assay errors during real time PCR, as a result, the relative transcription amount of different genes was individually obtained according to the standard curve of itself. 4-fold dilution gradient as well as 5 points were selected for making standard curve of each gene, and the efficiency was calculated by determining the gradient of the line through plots of Log_2_ ^(dilution factor)^ against the C_t_ values. For real time PCR, 16sr cDNA was used to correct the cell differences among all samples.

**The method for statistical analysis**

For the fermentations, the wild type and mutant strains were conducted with 3 [replication](http://dict.youdao.com/w/replication/)s, and the results showed in the current research are from the mean data with the good fermentation conditions. As for the RT-PCR, the analytic results were also performed with [experimental](http://dict.youdao.com/w/experimental/) [replication](http://dict.youdao.com/w/replication/) by at least three times.

# Supplemental Buffers and media

A buffer, 20 mM Tris-Cl, 150 mM NaCl, pH8.0

Binding buffer, 50 mM Tris-HCl pH7.6, 5 mM MgCl_2_, 1 mM dithiothreitol, 5% glycerol

Protein stock buffer, 50 mM Tris-Cl, 150 mM NaCl

Running buffer, 45 mM Tris-Cl (pH8.0), 45 mM boric acid, 1 mM EDTA and 2.5% glycerol

Sample buffer, 100 mM potassium phosphate, pH7.5, 20 mM EDTA, 10% acetone [vol/vol]

Stop solution, 3 M ammonium acetate, 0.25 M EDTA, 1 mg ml^-1^ glycogen

BPM21: [maltodextrin](http://dict.youdao.com/w/maltodextrin/) 80 g, Ca_2_CO_3_ 7 g, glucose 5 g, L-methionine 2 g, Filtered liquid of boiled Soy flour 1 L, pH 6.7.

# Supplemental tables

**Table S1 Strains and plasmids used in this study**

| **Strains, phages and plasmids** | **Relevant characteristics** | **Reference or source** |
| --- | --- | --- |
| ***Streptomyces* sp. NRRL 30471** |  |  |
| 30471 | Wild-type producer of muraymycin | NRRL |
| DM-5 | *mur34* mutant with the gene replaced by 1.5-kb of *neo* cassette of SuperCos1 in *Streptomyces* sp. NRRL30471. | This study |
| DM-6 | *mur33* mutant with the gene inframe-deleted in *Streptomyces* sp. NRRL30471. | This study |
| DM-7 | *mur32* mutant with the gene replaced by 1.5-kb of *neo* cassette from SuperCos1 in *Streptomyces* sp. NRRL30471. | This study |
| DM-14 | *mur34* mutation was complemented by the integration of pJTU5052 to the chromosome DNA of DM-5 | This study |
| WT/pJTU5034 | Wild type strain of *Streptomyces* sp. NRRL30471 containing pJTU5034 | This study |
| WT/pJTU5037 | Wild type strain of *Streptomyces* sp. NRRL30471 containing pJTU5037 | This study |
| WT/pJTU5038 | wild type strain of *Streptomyces* sp. NRRL30471 containing pJTU5038 | This study |
| WT/pJTU3700 | Wild type strain of *Streptomyces* sp. NRRL30471 containing pJTU3700 | This study |
| DM-5/pJTU5034 | DM-5 mutant containing pJTU5034 | This study |
| DM-5/pJTU5037 | DM-5 mutant containing pJTU5037 | This study |
| DM-5/pJTU5038 | DM-5 mutant containing pJTU5038 | This study |
| DM-5/pJTU3700 | DM-5 mutant containing pJTU3700 | This study |
| TK24/pJTU18F3 | *Streoptomyces lividans* TK24 containing 18F3 | This study |
| TK24/pJTU5642 | *Streoptomyces lividans* TK24 containing pJTU5642 | This study |
| TK24/pJTU5024 | *Streoptomyces lividans* TK24 containing pJTU5024 | This study |
| TK24/pJTU5053 | *Streoptomyces lividans* TK24 containing pJTU5053 | This study |
| ***E. coli***  DH10B | *F– mcrA ∆(mrr-hsdRMS-mcrBC) ф80d lacZ ∆M15 ∆lacX74 deoR recA1 endA1 ara∆139 D(ara, leu)1697 galU galKλ - rpsL nupG* | GIBCO BRL |
| BW25113 (pIJ790) | BW25113/pIJ790 K-12 derivative: Δ*araBAD* Δ*rhaBAD*/λ-Red (gam bet exo) *cat* *araC* rep101(Ts) | [1] |
| ET12567 (pUZ8002) | *dam dcm hsdS* pUZ8002 | [2] |
| BL21 |  | (Novagene) |
| ***Bacillus subtilis*** | Indicator strain sensitive to muraymycins | (Lab collection) |
| **Plasmids/cosmid** |  |  |
| pOJ446 | *aac(3)IV,* SCP2, *rep*^pMB1*^, *attФC31*, *ori*T | [3] |
| pSET152 | *aac(3)IV*, *lacZ*, *rep*^pMB1*^ *attФC31*, *ori*T | Bierman *et al* |
| pET28a | *neo*, *lacI*, *ori*f1, T7 lac, pBR322 origin, His_6_∙tag | (Novagene) |
| pBluescript II SK (+) | *bla*, *lac*Z, *ori*f1 | [4] |
| pJTU3700 | Derivative of pSET152 with *xylE* | (Dai *et al*, unpublished) |
| pJTU2463 | Derivative of pOJ446 with SCP2 replicon replaced by *int* and *attp* from pSET152 | (Yao *et al.* unpublished) |
| pJTU2463b | Derivative of pJTU2463 with XbaI and SpeI sites blocked | (Chen *et al.* unpublished) |
| pJTU5629 | pBluescript II SK (+) derivative with a *ca*. 14.6-kb ScaI fragment (Fig. 2) from cosmid 18F3 cloned into the EcoRV site of the vector | This study |
| pJTU5633 | pOJ446 derivative with *ca*. 14.6-kb ScaI fragment (Fig. 2) from cosmid 18F3 cloned into the EcoRV-SpeI sites of the vector | (Cheng *et al*, unpublished) |
| pJTU5634 | pJTU5633 derivative with *neo* cassette inserted into *mur34* | This study |
| pJTU5642 | 18F3 derivative with *neo* cassette inserted into *mur34* | This study |
| pJTU5030 | pJTU5642 derivative with *neo* cassette deleted | This study |
| pJTU5020 | pOJ446 derivative with left (1146-bp) and right (1355-bp) homologous arms of *mur33* cloned into the XbaI-SpeI sites of the vector | This study |
| pJTU5034 | pJTU3700 derivative with 351-bp upstream and 365-bp downstream of the transcription start point of *mur33* inserted into, upstream of the *xylE* gene | This study |
| pJTU5036 | pET28a derivative with *mur34* cloned into the NdeI-BamHI sites of the vector | This study |
| pJTU5037 | pJTU3700 derivative with *ca.* 0.7-kb fragment of *mur33* promoter sequence with six base pairs of -10 region mutation inserted into the upstream of *xylE* gene | This study |
| pJTU5038 | pJTU3700 derivative with *ca.* 0.7-kb fragment of *mur33*promoter sequence with seven base pairs of -35 region mutation inserted into the upstream of *xylE* gene | This study |
| pJTU5039 | pJTU5633 derivative with *neo* cassette inserted into *mur32* | This study |
| 18F3 | cosmid include muraymycin gene cluster from *mur6* to *mur38* | [5] |
| pJTU5052 | *mur34* with its own promoter 224-bp upstream was ligated to pSET152 | This study |
| pJTU5024 | pJTU5030 derivative with *neo* cassette inserted into *mur11* | This study |
| pJTU5053 | pJTU5030 derivative with inframe-deleted of *mur12* | This study |

**Table S2 Primers used in this study**

| primers | Sequence(5’-3’) | Note |
| --- | --- | --- |
| mur11-F | GAATCGCTCGCGAAGGAG | RT-PCR primers |
| mur11-R | CACTGGTAGCGCTTCTGG |  |
| mur12-F | CCTGGTGGACTCCGTCG |  |
| mur12-R | TCGAACTCGGTGTCCGG |  |
| mur27-F | AAGAGAACCGCGGGGGA |  |
| mur27-R | ATCTGCGGCAGCGTGAG |  |
| mur28-F | GGTCCTTTCGGTAGCGG |  |
| mur28-R | CCACCATGTCACGCACC |  |
| mur33-F | TGCGCAGCCCGACAGAT |  |
| mur33-R | CCGTATGCGCTGGAAGC |  |
| mur17-F | TACGGCCCCGGTCGCTG |  |
| mur17-R | GCGGTTCTCGCTCGGTAC |  |
| 16-F | AGTAACACGTGGGCAACTGC |  |
| 16-R | CTCAGACCAGTGTGGCCGGT |  |
|  |  | *Mur11*, *mur12*, *mur32*, *mur33* and *mur34* mutation related primers |
| Mur11F | TTCGAATTCCGGAACTGTCAGAGCCCGGAGCACTCGAAGAGGTTCTAGAGCTATTCCAGAAGTAG |  |
| Mur11R | GCGTGGGAGCCGGAACCATCCCCAGCTACCCGGTGCATGCACGACTAGTCTGGATGCCGACGG |  |
| mur11-det-F | GGAATTCCATATGGTCCAGCTGAACATCAC |  |
| mur11-det-R | CGGGATCCTTAAGCCTTTTCCACCAGAGC |  |
| Mur12F | AGCGAACTGTCCGCCAAGGTGGATGCTCGGGTCGCCGAACTCACTTCTAGATATTCCAGAAGTAG |  |
| Mur12R | CGATTTCTTCCAGCTCAATCCGCTGACCGTGCAGCTTGATCTGGGACTAGTCTGGATGCCGACG |  |
| Mur12-det-F | TACCATATGAGTCAGGCCCCGGTACG |  |
| Mur12-det-R | ATCGAATTCTCACGCCTCCGGACCGGACG |  |
| mur34F | GAGGCCCTCTATCCCGATGAGCGCACCCGTCCGGGGTACTCTAGAGCTATTCCAGAAGT |  |
| mur34R | ACTGGCAGGCGACAGCCCGTCAGCGCGAAGGGCTATGCTACTAGTCTGGATGCCGACG |  |
| mur34-det-F | GGAATTCCATATGCCCAATGGTGCAG |  |
| mur34-det-R | CGGGATCCCTATTCGGGGTTCTCG |  |
| mur34-selfF | CGGGATCCAGCGCTGACCGGCTCCCC |  |
| mur34-selfR | CCGATATCCTATTCGGGGTTCTCGGTGG |  |
| mur32F | CGCCGCCGTCAGGGGGCGGCAGCCGATCGCGGCCCCTGCTCTAGAGCTATTCCAGAAGT |  |
| mur32R | GCGTCCGCTCGACGCTGAGGACGGAGTAGCCGTGTGAGGACTAGTCTGGATGCCGACG |  |
| mur32-det-F | ATTCATATGAACATGCGATCCGTTCCC |  |
| mur32-det-R | TATAAGCTTTCACTCCGGCCGGACCGG |  |
| mur33-EX-F1 | GCTCTAGAGTCGGCGAATTCGTGG |  |
| mur33-EX-R1 | CCGCTCGAGGACAACGAAATCGTCGAG |  |
| mur33-EX-F3 | CCGCTCGAGGATGTTCTCCTCGCGCA |  |
| mur33-EX-R2 | GGACTAGTCTTGCGGAATTCTTCGG |  |
| Gsp1-1 | CTGGTCGGGGAGAATGAT | 5’ RACE primers |
| Gsp2-1 | CGGCCCGGCGACCTGGTAA |  |
| Gsp3-1 | CGACGATATAGCTCGCCGTGGGAT |  |
| AAP | GGCCACGCGTCGACTAGTACGGGIIGGGIIGGGIIG |  |
| AUAP | GGCCACGCGTCGACTAGTAC |  |
| orf15/16-F | GACCGAACCGAGCAGTCGAT | PCR primers to amplify the regions between *mur* genes |
| orf15/16-R | AGTCAGCGCGGACCACGTTC |  |
| orf32/33-F | GCGCGTCGAGGGACAGGGCC |  |
| orf32/33-R | CTCCTGCCGCCGGTCAAAGC |  |
| orf33/34-F | TAGTTAACAAATGGCCACTCGGCG |  |
| orf33/34-R | ACAACCCCAATCCAACCGCG |  |
| orf29/30-F | GGCGCAGGGCCACGTTG |  |
| orf29/30-R | TCATCGGTCCTCGCGGC |  |
| orf20/21-F | CTTCGCTGATGGCCACAC |  |
| orf20/21-R | CTTCCAGGGCGAGCGCA |  |
| orf8/9-F | TTTGTGTCCAGGGCTCGG |  |
| orf8/9-R | TTGCGTGCATGGTCCTGG |  |
| orf10/11-F | CGGCACCTTCGCCCGCTT |  |
| orf10/11-R | GCTCTGGTGGAAAAGGCTTAA |  |
| orf11/12-F | ATGAACTCGGCGTGCGCGAT |  |
| orf11/12-R | ACCGCGCAAAGGCTGTCCAC |  |
| orf13/14-F | CGCTGCGCCTGGACGGTG |  |
| orf13/14-R | CCTCGACAAAGAAGTTGTCGT |  |
| orf24/25-F | GGGCCAGGCCGGGCTCAA |  |
| orf24/25-R | GCCTCGCAGCAGCCAGCAT |  |
| orf25/26-F | ACATGCTCGCCGTACACCTG |  |
| orf25/26-R | GACGACGCACTCGATGCTC |  |
| orf26/27-F | GGCGCGTCGGCGACGAG |  |
| orf26/27-R | GGCGACGATCTGCGGCAG |  |
| orf27/28-F | CTGCGCGCCCACCCTGAC |  |
| orf27/28-R | GGGTCGTCTTACCGCTACC |  |
| orf28/29-F | TGCGAGCATGGCGACTGGC |  |
| orf28/29-R | TACAACCCCTCGACGAGCC |  |
| orf30/31-F | GAGGACGGTTCGCCCGATC |  |
| orf30/31-R | GCACCCAGGCTGGCCAGC |  |
| orf31/32-F | CTTCGCCGGGATGTTCGTG |  |
| orf31/32-R | CTACCGTGTACGTGCCGGC |  |
| orf34/35-F | CGGGGTTCTCGGTGGCATCC |  |
| orf34/35-R | CTGACCGGCTCCCCTCATCA |  |
| orf35/36-F | GTTCGCCGTCAAATGGGTGG |  |
| orf35/36-R | CCTGATGGCCACCGCCTC |  |
| orf36/37-F | GCTGCCTCCGACTGTAACGA |  |
| orf36/37-R | GATTGCTCGACCATGACCGT |  |
| mur10-PF | GCCTCCGATGTGAGGGA | Gel-shift primers |
| mur10-PR | GCTCTGGTGGAAAAGGCT |  |
| mur11/12-PF | TTCGGTCGATCAGTCATCG |  |
| mur11/12-PR | AGGCTGTCCACGAGCCG |  |
| P12-Fam-F | AGCCAGTGGCGATAAGTTCGGTCGATCAG |  |
| mur33-PF | ACTACTTGTTCACAACAGCG |  |
| mur33-PR | GGCTCCCGACCCTTGGA |  |
| mur33PR11 | GAGTGGCCATTTGTTAACTATA |  |
| mur33-2F | AGCGGATGCTGCATCCCGC |  |
| mur33-9R | GAAATCGTTGGCACATGAGGG |  |
| mur34-PF | GAGCCAGCCTCACTTAGG |  |
| mur34-PR | AGCGCTGACCGGCTCC |  |
| mur36-PF | TGCCTCCGACTGTAACGA |  |
| mur36-PR | GCCGGTTGAGGTTGGCT |  |
| mur33-P-PF | CCCCGCGGGACCCTTGGACCCTGAGGCC | *mur33* promoter primers |
| mur33-P-PR | CGGGATCCGATGTTCTCCTCGCGCAGGG |  |
| M13F | TGTAAAACGACGGCCAGT |  |
| M13R | CAGGAAACAGCTATGACC |  |
| P33-10F | TAACAAATGGCCACTCGGCG |  |
| P33-10R | ATAGAACGCTGGGTTGACAAG |  |
| P33-35F | ACGCGAGCCACGAGCATG |  |
| P33-35R | TCGCCGAGTGGCCATTTGT |  |

**Table S3 Antibiotics**

| Antibiotics | amplicilin | apramycin | chloramphenicol | kanamycin | Nalidiaxic acid |
| --- | --- | --- | --- | --- | --- |
| Concentration/μg ml^-1^ | 100 | 50 | 34 | 50 | 25 |

# Reference

1. Gust B, Challis GL, Fowler K, Kieser T, Chater KF (2003) PCR-targeted Streptomyces gene replacement identifies a protein domain needed for biosynthesis of the sesquiterpene soil odor geosmin. Proc Natl Acad Sci U S A 100: 1541-1546.

2. Paget MS, Chamberlin L, Atrih A, Foster SJ, Buttner MJ (1999) Evidence that the extracytoplasmic function sigma factor sigmaE is required for normal cell wall structure in Streptomyces coelicolor A3(2). J Bacteriol 181: 204-211.

3. Bierman M, Logan R, O'Brien K, Seno ET, Rao RN, et al. (1992) Plasmid cloning vectors for the conjugal transfer of DNA from Escherichia coli to Streptomyces spp. Gene 116: 43-49.

4. Short JM, Fernandez JM, Sorge JA, Huse WD (1988) Lambda ZAP: a bacteriophage lambda expression vector with in vivo excision properties. Nucleic Acids Res 16: 7583-7600.

5. Cheng L, Chen W, Zhai L, Xu D, Huang T, et al. (2011) Identification of the gene cluster involved in muraymycin biosynthesis from Streptomyces sp. NRRL 30471. Mol Biosyst 7: 920-927.
