# Supplementary material for: Identification of Mur34 as the Novel Negative Regulator Responsible for the Biosynthesis of Muraymycin in Streptomyces sp. NRRL30471
Source: PLoS One. 2013 Oct 15;8(10):e76068. doi: 10.1371/journal.pone.0076068 (PMC3797123; doi:10.1371/journal.pone.0076068)
Supplement: Figure S1 — In silico analysis of Mur34 and Mur33. (A) Analysis of Mur34 with its homologous proteins was performed by clustal W software. The secondary structure of Mur34 was analyzed by SWISS-MODEL online program (http://swissmodel.expasy.org/). (B) The secondary structure of Mur33 C-terminal was analyzed by SWISS-MODEL, and the homologous proteins from the biosynthetic pathways of translocase I inhibitors were analyzed by clustal W. The red boxes showed the a-helix domains which contribute to the DNA-binding activity. (PDF) [file pone.0076068.s001.pdf]

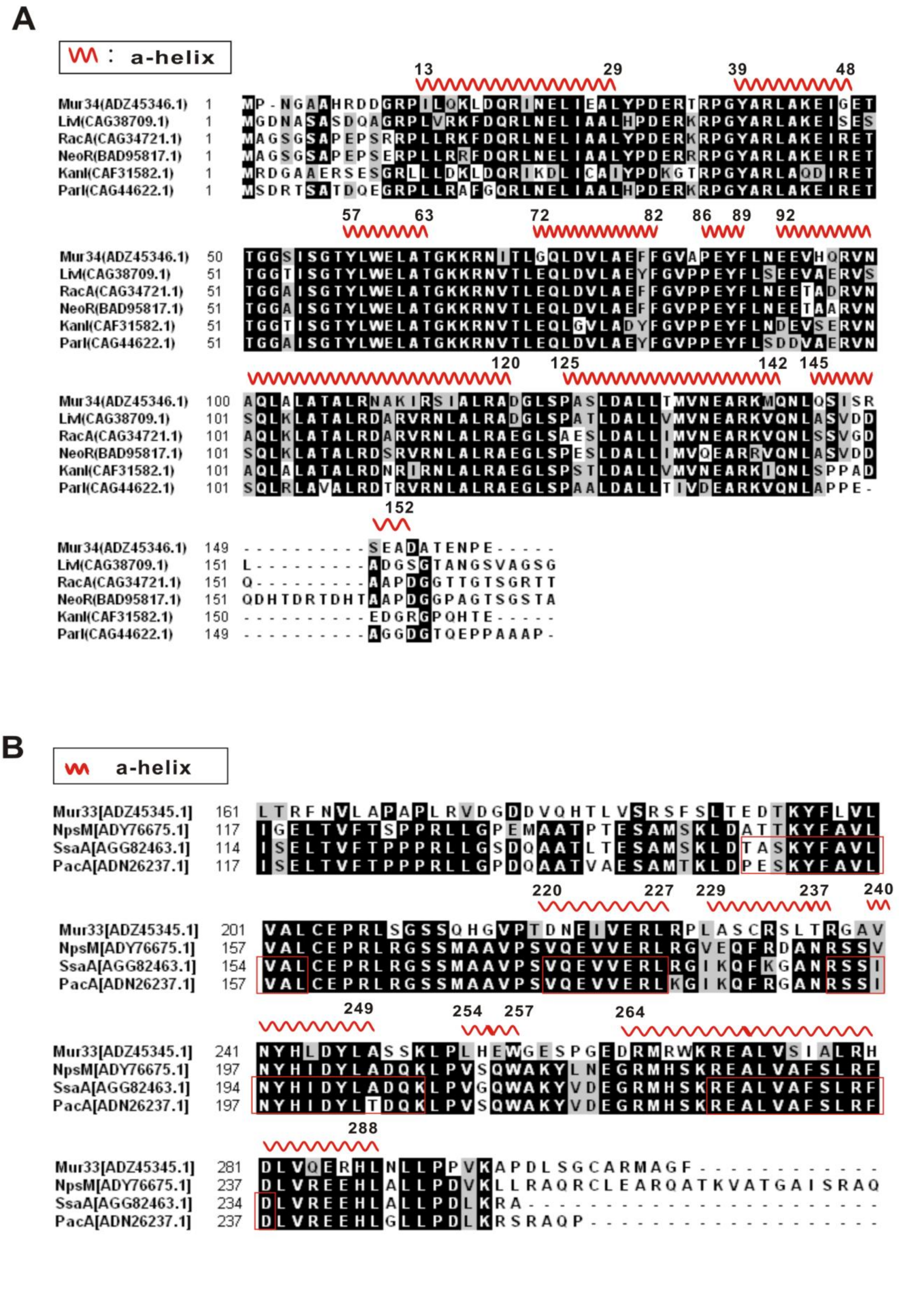

A
a-helix
Mur34(ADZ45346.1)
LiM(CAG38709.1)
RacA(CAG34721.1)
NeoR(BAD95817.1)
KanI(CAF31582.1)
ParI(CAG44622.1)
B
a-helix
Mur33[ADZ45345.1]
NpsM[ADY76675.1]
SsaA[AGG82463.1]
PacA[ADN26237.1]
